# Genetic and Biological Properties of an Epidemic Feline Panleukopenia Virus Strain (Ala91Ser) in China

**DOI:** 10.3390/vetsci12070668

**Published:** 2025-07-16

**Authors:** Erkai Feng, Zihan Ye, Manping Yan, Yaxi Zhou, Danni Wu, Shipeng Cheng, Yuening Cheng

**Affiliations:** Key Laboratory of Economic Animal Diseases, Ministry of Agriculture, Institute of Special Animal and Plant Science, Chinese Academy of Agriculture Science, Changchun 130011, China; fengerkai@aas.cn (E.F.); youzi0915ye@163.com (Z.Y.); yanmanping310@163.com (M.Y.); 13001757157@163.com (Y.Z.); 13321435868@163.com (D.W.)

**Keywords:** Ala91Ser, epidemic, feline panleukopenia virus, immunogenicity, pathogenicity

## Abstract

To genetically characterise an epidemic isolate of feline panleukopenia virus (FPLV) harbouring the Ala91Ser mutation in China, a clinical strain (accession number: OR921195.1), named FPLV-CC19-02, was isolated from a PCR-positive faecal swab sample. Phylogenetic analysis revealed that it is far removed from all current commercial vaccine strains and differs from the FPLV prototype strain Cu-4 (M38246.1), specifically the vaccine strain of Fel-O-Vax^®^ PCT, at positions 91 (Ala91Ser) and 101 (Ile101Thr) within the VP2 protein. This virus can induce the typical cytopathic effect seen in parvovirus infection in feline kidney cells, resulting in severe clinical symptoms in cats, including haematochezia and hyperthermia. Furthermore, infected cats died of virus infection within 5–10 days post-infection (dpi) (100% morbidity and 83% mortality), indicating that FPLV-CC19-02 is a strain with increased virulence. Additionally, it demonstrated good immunogenicity in cats. Overall, these findings may help us to better understand the molecular prevalence of feline panleukopenia virus in cats and provide valuable basic data for the development of effective, locally adapted feline panleukopenia virus vaccines in China.

## Simple Summary

A Chinese epidemic feline panleukopenia virus variant, named FPLV-CC19-02, was isolated from a PCR-positive faecal swab sample in this study. It shows a long distance to from all current commercial vaccine strains (Pfizer^®^, New York, NY, USA; MSD^®^, Kenil-worth, NJ, USA; Virbac^®^, Nice, Alpes-Maritimes, France; Merial^®^, Paris, France; Nobivac^®,^ Boxmeer, Feldhoven, The Netherlands) in the phylogenetic tree, and differs from the FPLV prototype strain Cu-4 (M38246.1) at the 91st (Ala91Ser) and 101st (Ile101Thr) site within the VP2 protein. Additionally, it exhibits more virulence for host cells and cats. Meanwhile, the vaccination of cats against FPLV infection demonstrated good immunogenicity result in cats. These findings suggest that this variant is a better candidate for the development of a locally produced FPLV vaccine in China.

## 1. Introduction

Parvovirus infection in cats has been recognised as a disease for over 100 years [1,2] and was once known as feline distemper [3,4], feline infectious enteritis [5,6], or feline enteritis [3], in many early studies. However, it is now commonly referred to as feline panleukopenia.

Feline panleukopenia (FPL), primarily caused by the feline panleukopenia virus [7], is a highly contagious and often fatal viral disease of cats. All members of the cat family (Felidae) are affected, as well as a few members of the Procyonidae family (suborder Caniformia) and the weasel family (Mustelidae), such as the raccoon [8], mink [9], and even some rare and endangered wild animals, like the captive giant panda [10,11] and the Siberian tiger [12]. Infected animals typically present with lethargy, anorexia, vomiting, and diarrhoea, followed by progressive dehydration, marked leukopenia, damage to the intestinal mucosa, and, ultimately, death [13].

Feline panleukopenia virus (FPLV), also known as feline parvovirus [14,15], is a small, non-enveloped, single-stranded DNA virus that belongs to the Parvoviridae family, specifically the Parvovirus genus. It was first identified as the viral cause of FPL in the 1920s [2] and was isolated via tissue culture in 1965 [16]. The FPLV genome is approximately 5000 base pairs (bp) in length and contains two open reading frames (ORF1 and ORF2), which encode the non-structural proteins (NS1 and NS2) and the structural proteins (VP1 and VP2), respectively [17].

The VP2 protein is the major structural component of FPLV and is critically involved in determining host specificity and inducing protective, neutralising antibodies during infection [18]. CPV-2 is believed to have emerged from FPLV, based on several missense mutations at site 80 (Lys-Arg), 93 (Lys-Asn), 103 (Val-Ala), 232 (Val-Ile), 323 (Asp-Asn), 564 (Asn-Ser), and 568 (Ala-Gly) [19,20]. In recent years, FPLV strains isolated from giant pandas possess a Gly299Glu substitution in VP2 [10], whereas those derived from dogs typically harbour a Thr101Ile mutation [17]. Furthermore, the Ala300Pro mutations may facilitate the expansion of host cell tropism, enabling the FPLV variant to replicate efficiently in canine-derived cells [21].

Since 2017, a FPLV variant carrying an Ala-Ser mutation at the 91st site of the VP2 sequence has emerged and has rapidly become pandemic among cats in China, resulting in higher morbidity and mortality. Additionally, Vietnam appears to have experienced an outbreak of the same pandemic, caused by the FPLV variant (Ala91Ser) infection. Therefore, greater attention should be given to this variant.

In this study, we describe the isolation and identification of the Ala91Ser FPLV variant, its phylogenetic relationship, and its pathogenicity and immunogenicity in cats. Our findings aim to provide valuable data for controlling this sudden pandemic disease in cats and for the development of an effective, locally adapted vaccine in China.

## 2. Materials and Methods

### 2.1. Samples, Cells, and Cats

A faecal swab collected from a pet hospital in the Chaoyang district of Changchun city, Jilin province, and previously identified as FPLV-positive through a triple nanoPCR assay [22], was utilised in this study. Feline kidney cells (F81 cells), obtained from the Cell Resource Centre of the Shanghai Institute of Biological Sciences, Chinese Academy of Sciences, were cultured in minimum essential medium (Gibco, Waltham, MA, USA) containing 10% foetal bovine serum (Gibco, Waltham, MA, USA), 2 mM L-glutamine (TransGen Biotech, Beijing, China), and 1% penicillin-streptomycin (100 U/mL; Thermo Fisher, Waltham, MA, USA). The cells were incubated at 37 °C in a 5% CO_2_ environment.

Nineteen British shorthair cats, aged 8 to 10 weeks and with haemagglutination inhibition (HI) titres below 1:8, were acquired from Jintai Meidi Biotechnology Co., Ltd. (Changchun, Jilin, China). These cats were acclimatised in a controlled environment and employed in the studies when they reached 10 to 12 weeks of age.

### 2.2. Virus Isolation

The virus was isolated using a method previously described [23]. Briefly, faecal swabs from FPLV-positive cats were homogenised in serum-free MEM medium at a volume-to-volume ratio of 1:9. After undergoing three cycles of freezing and thawing, the supernatant was collected by centrifugation at 10,000 g for 30 min at 4 °C (rotator-type F-34-6-38, Eppendorf, Hamburg, Germany). Subsequently, the supernatants were filtered using a 0.22 μm needle filter. Then, the F81 cells were inoculated with the FPLV-positive supernatant and maintained at 37 °C in a CO_2_ incubator for 4–5 days. The cytopathic effect (CPE) of the infected F81 cells was monitored daily. Cell cultures exhibiting CPE were stored at −80 °C.

The hemagglutination of the isolated virus was assessed using a hemagglutination assay (HA). Briefly, the isolated FPLV was serially diluted in a phosphate-buffered saline solution (PBS) at pH 6.5 (0.5% bovine serum). Two-fold serial dilutions of the virus in PBS were prepared, ranging from 1:2 to 1:2048. Twenty-five microliters of each dilution were mixed with an equal volume of PBS, and the plate was incubated at 37 °C for 60 min. Two volumes of a 1% porcine erythrocyte suspension were added to each well, and the plate was then incubated at 4 °C for 40 min. The HA titre was determined as the reciprocal of the highest FPLV isolate dilution exhibiting an HA reaction.

### 2.3. Direct Immunofluorescence Assay

A direct immunofluorescence assay was performed to identify the virus. Briefly, F81 cells infected with the virus for 2 days in 6-well plates were fixed with pre-cooled acetone for 45 min at 4 °C. Subsequently, the cells were incubated with 1% bovine serum albumin (BSA) for 1 h at 37 °C to block non-specific protein-protein interactions. The cells were then incubated with a fluorescein isothiocyanate (FITC)-conjugated monoclonal antibody (Mab) specific for FPLV (VMRD, Inc., Pullman, WA, USA) for an additional hour at 37 °C. Finally, the cells were observed under a fluorescence microscope (Leica AF 6000, Wetzlar, Germany).

### 2.4. Transmission Electron Microscopy (TEM)

The morphology of the isolated viruses was examined using transmission electron microscopy (TEM) following negative staining, as previously described [11].

Virus samples were also purified by centrifugation, collected in PBS (phosphate-buffered saline), and negatively stained with 2% phosphotungstic acid to observe virus particles under TEM (JEOL JEM-1400 FLASH, Tokyo, Japan).

### 2.5. PCR Sequencing and Phylogenetic Analysis

Viral DNA was extracted from the virus isolates using the Takara MiniBEST Viral RNA/DNA Extraction Kit, Version 5.0 (Takara, Beijing, China), following the manufacturer’s instructions. PCR amplification of the full-length VP2 gene was performed using a set of primers: VP2-F: 5′-CTTACGCTGCTTATCTTCGCTCTGG-3′ and VP2-R: 5′-TTTTGGTCCTTAACATATTCTAAGGGC AA-3′ [24]. The standardised PCR system contained 3 μL of viral DNA, 25 μL of Prime STAR^®^ Max DNA Premix (2X) (Takara, Beijing, China), 1.5 μL each of the primers, and 19 μL of sterile distilled water. The PCR was performed with the following steps: initial denaturation at 98 °C for 120 s, followed by 35 cycles of denaturation at 98 °C for 15 s, annealing at 58 °C for 15 s, and extension at 72 °C for 150 s, with a final extension at 72 °C for 600 s. The PCR products were analysed by electrophoresis using a 1% agarose gel and then sequenced using an ABI 3730XL DNA analyser from Comate Bioscience Co., Ltd. (Changchun, Jilin, China).

The obtained sequences (accession number: OP471917.1) underwent BLASTn analysis to ascertain the homology percentage of our isolate with the other available isolates in GenBank. A phylogenetic tree was constructed using MEGA 11 (State College, PA, USA) to compare our isolate with the available VP2 gene sequences of various FPLV isolates. The maximum likelihood method was employed with 500 bootstrap replicates. A total of 100 nucleotide sequences were retrieved from GenBank and incorporated into the dataset (Table S1 in the Supplementary Materials).

### 2.6. Screening the A91S FPLV Variant in the GenBank Database

To gain a deeper understanding of the epidemiologic features of the Ala91Ser FPLV variant globally, 770 complete VP2 sequences of FPLV that were available in the GenBank database (up to 13 April 2024) were screened based on the VP2 amino acid residue at position 91. The corresponding information regarding the country, host, collection date, and number of Ala91Ser FPLV isolates per year among all FPLV isolates was recorded (Table S4 in the Supplementary Materials).

### 2.7. Pathogenicity Studies of the A91S FPLV Variant in Cats

Nine British shorthair cats with HI titres of less than 1:8, housed in a single animal facility, were divided into two groups: six cats were in the experimental group (with the assigned ID numbers 4258,4260, 4402, 4544, 4243, and 4241) and three were in the control group (with the assigned ID numbers 4254, 4257, and 4406). The cats in the experimental group were orally challenged with 5 mL of cell culture containing the isolated virus (FPLV-CC19-02, HA = 1024, TCID_50_ = 10^6.5^/mL). In contrast, those in the control group received the same dose of MEM. The treated cats were monitored for clinical symptoms (body temperature, faecal consistency, and appetite) over 10 days. To collect the baseline of data, measurement was conducted on the day before inoculation. The clinical symptoms and grading criteria are shown in Table 1. White blood cell (WBC) counts were obtained at 1-, 3-, 5-, and 7-days post-inoculation (dpi) using the VetScan HM5 haematology analyser (Zoetis, Union City, CA, USA). Additionally, their faeces were tested daily for virus shedding using FPLV antigen rapid test immune-chromatographic strips produced by SHANGHAI QUICKING Biotech CO., Ltd. (Shanghai, China). One cat in the control group was euthanised via an intravenous injection of propofol (0.6 mg/kg, Jiabo Co., Ltd., Guangzhou, China) and potassium chloride solution (100 mg/kg, MACKLIN Co., Ltd., Shanghai, China) to avoid unnecessary suffering. The spleen, kidneys, mesenteric lymph nodes, small intestine, and large intestine were collected from the euthanised and dead cats and were then used in follow-up studies.

### 2.8. Virus Distribution in Tissue Organs

Two pairs of primers were designed based on the VP2 sequence of the FPLV variant (OP471917.1) and the glyceraldehyde-3-phosphate dehydrogenase (GAPDH) sequence: FPLV-F: 5′-GAAGCGTCTACACAAGGGC-3′, FPLV-R: 5′-CTCTCAGGTGTTTCTCCTGTTG-3′; GAPD H-F: 5′-ACCATCTTCCAGGAGCGAGAT-3′ and GAPDH-R: 5′-ATGATGACCCTCTTGGCCC-3′. These primers amplify the products of 155 bp and 141 bp, respectively.

The assays were performed on an ABI 7500 instrument (Applied Biosystems Inc., Waltham, MA, USA) under the following reaction and cycling conditions: the reaction volume was 10 µL, consisting of 5 µL of 2x SYBR^®^ Green PCR Master Mix (QIAGEN, Dusseldorf, Germany), 0.05 µL of ROX^TM^ Reference Dye, 0.7 µL of each primer for VP2 or GAPDH, 1 µL of template gDNA or cDNA, and RNase-free water, which was added to achieve the final volume. The reaction conditions were as follows: 120 s at 95 °C, followed by 40 cycles of 5 s at 95 °C and 30 s at 60 °C.

All samples (spleen, kidney, mesenteric lymph node, small intestine, and large intestine) from one animal were tested for the presence of the VP2 gene and the GAPDH gene using the same real-time quantitative PCR (RT-qPCR) method. Each sample was tested three times, and a template control was included to verify the accuracy of the DNA extraction process.

### 2.9. Immunogenicity Study of Ala91Ser FPLV Variant in Cats

The killed FPLV vaccine was prepared by mixing the SEPPIC SA 50 adjuvant (Seppic, Shanghai, China) with an inactivated virus solution (HA = 1024, TCID_50_ = 10^6.5^/mL) at a ratio of 1:9. The Ala91Ser FPLV virus culture was inactivated using a binary ethylenimine (BEI) solution at a final concentration of 0.002 mol/L. The residual BEI was neutralised by adding a sodium thiosulfate solution to a final concentration of 0.002 mol/L.

Ten British shorthair cats, with HI titres of less than 1:8, were randomly divided into two groups: Group I (five kittens) received the killed FPLV vaccine, and Group II (five kittens) received Fel-O-Vax^®^ PCT (Zoetis, Suzhou, China). All cats were vaccinated subcutaneously with a single dose (1.0 mL). Blood samples were collected from all cats at 0-, 7-, 14-, and 21 days post-vaccination (dpv). The serum antibody titres against FPLV were determined using a HI assay, which was conducted with 1% pig erythrocytes and 4 HA units. The HI titres were calculated as the reciprocal of the highest serum dilution that completely inhibited hemagglutination. Each serum sample was tested in triplicate.

### 2.10. Statistical Analysis

Analysis of the data was performed using GraphPad Prism software (version 9.0, San Diego, CA, USA). The data were represented as mean ± SD (standard Deviation). Significant differences were analysed using Student’s *t*-test. A score of *p* < 0.05 was considered statistically significant (*), while *p* < 0.01 was considered statistically highly significant (**).

## 3. Results

### 3.1. Isolation and Identification of Ala91Ser FPLV Variant

**Figure 1:**
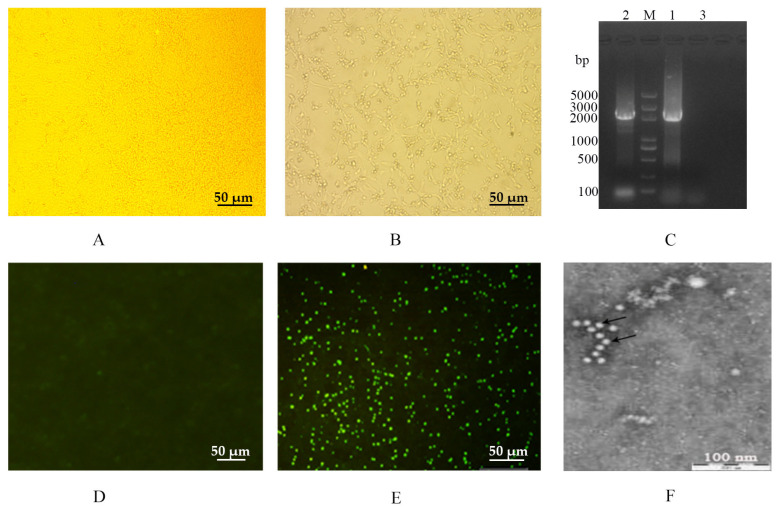
Isolation and identification of the FPLV epidemic strain (FPLV-CC19-02) in China. (**A**,**D**): an F81 cell; (**B**,**E**): an F81 cell infected with FPLV-CC19-02; (**C**): amplification of the full-length VP2 gene of FPLV-CC19-02. DNA 5000 ladder; lane 1: FPLV-CC19-02; lane 2: Fel-O-Vax^®^ PCT; lane 3: nuclease-free water; (**F**): morphology of FPLV-CC19-02 under electron microscopy.

After three rounds of blind passage, the FPLV-positive supernatant effectively induced normal F81 cells (Figure 1A) to exhibit the typical cytopathic effects (CPE) of parvovirus between 3–5 dpi. These effects were characterised by cell rounding, floating, pyknosis, disruption, and eventually necrosis (Figure 1B).

After PCR amplification, a gene fragment of approximately 2366 bp in size, covering the full length of the VP2 gene, was obtained (Figure 1C, lane 1) and further sequenced by Comate Bioscience Co., Ltd. (Changchun, Jilin, China).

The direct immunofluorescence assay indicated that the isolated virus (named FPLV-CC19-02) could react with the FITC-conjugated Mab of FPLV (VMRD, Inc., Pullman, USA). It showed bright green fluorescence in virus-infected F81 cells (Figure 1E). The HA assay results showed that the virus displayed hemagglutination activity as high as 1:1024.

Under a transmission electron microscope, spherical virus particles with diameters of 20–25 nm were observed in an isolated virus cell culture (P4) stained with 2% phosphotungstic acid. These observations align with the structural characteristics of typical parvoviruses (Figure 1F).

### 3.2. Phylogenetic Analysis of the Ala91Ser FPLV Variant

The obtained VP2 gene sequence of the Ala91Ser FPLV variant (accession OP471917.1) exhibited 100% nucleotide identity to 12 Chinese FPLV strains, including canine parvovirus (MT179777.1), and Panthera leo parvovirus Z30 (OP745049.1). Additionally, it shows 99.83–99.94% nucleotide identity to four foreign FPLV isolates (EU252146.1, HQ184195.1, EU252147.1 and EU498699.1) (Table S1 in the Supplementary Materials).

**Figure 2:**
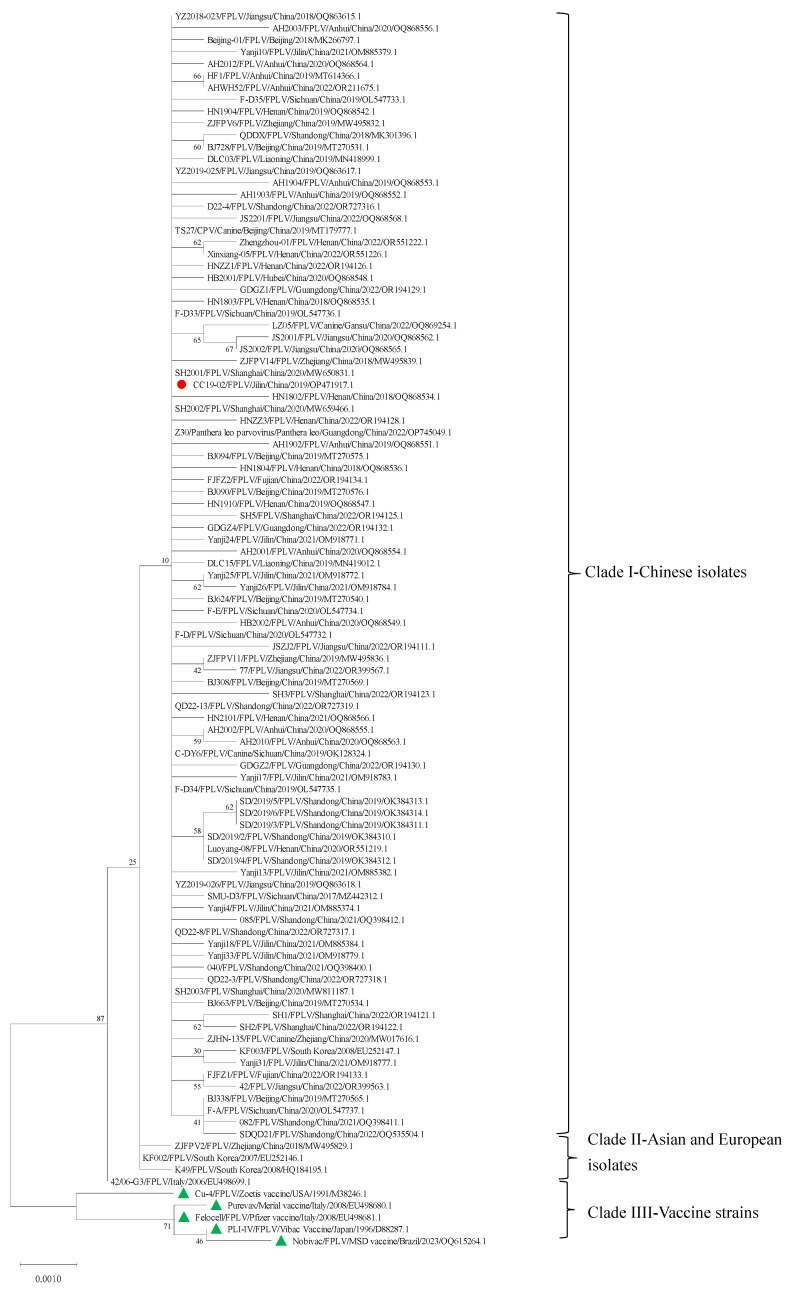
Phylogenetic analysis of FPLV-CC19-02.

The Ala91Ser FPLV variant showed the closest relationship with some Chinese FPLV isolates collected from the Shanghai municipality (MW65083.1, MW659466.1), Henan province (OQ868534.1), and Zhejiang province (MW495839.1) (Figure 2, Clade I). Conversely, it shows a distant relationship with isolates from South Korea (EU252146.1, HQ184195.1) and Italy (EU498699.1) (Figure 2, Clade II), and shows a further distance from commercial vaccine strains (M38246.1, EU498680.1, EU498681.1, D88287.1, and OQ615264.1) (Figure 2, Clade III).

Further analysis indicated that there are eight synonymous mutations (G171A, T300C, T663C, C810T, T871C, G1041A, G1521A, and T1572C) and four non-synonymous mutations (G271T, T302C, A694G, and C1684G) in the VP2 sequences among FPLV-CC19-02, five vaccine strains, and four Asian-European isolates (Table 2, Tables S2 and S3 in the Supplementary Materials). At the amino acid level, four non-synonymous mutations result in four significant amino acid changes at positions 91 (Ala91Ser), 101 (Ile101Thr), 232 (Ile232Val), and 562 (Leu562Val) (Table 3 and Table S3 in the Supplementary Materials).

### 3.3. Epidemiological Investigation of the Ala91S FPLV Variant

**Figure 3:**
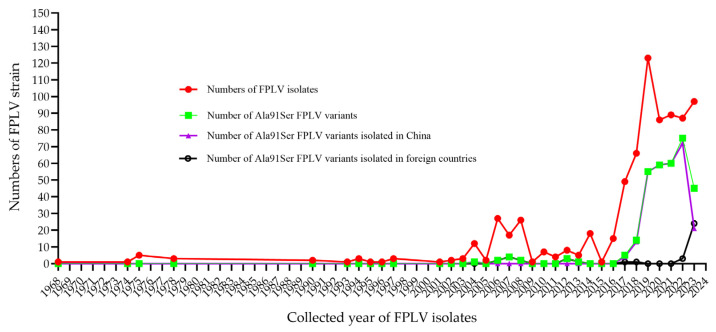
Epidemiology investigation of Ala91S FPLV variants around the world.

After screening the NCBI database, we found that the Ala91Ser mutation was first identified in a tiger-derived FPLV (DQ099431, Table S4 in the Supplementary Materials) in China in 2004. Subsequently, the Ala91Ser FPLV variant was sporadically identified in Italy, Hungary, and South Korea between 2006 and 2007. Through a retrospective study, some Ala91Ser FPLV variants were identified fragmentarily in Brazil since 2008. Interestingly, the mutation re-emerged in China in 2017, without any apparent reason. The proportion of Ala91Ser FPLV variants among the total number of FPLV virus strains reported annually in China increased from 10% in 2017 to 87% in 2022 and 2023 (Figure 3 and Table S4 in the Supplementary Materials). Additionally, an increasing number of Ala91Ser FPLV variants have been reported in Vietnam since 2023, suggesting a potential regional outbreak in Southeast Asia.

### 3.4. Pathogenicity Studies of the Ala91S FPLV Variant in Cats

**Figure 4:**
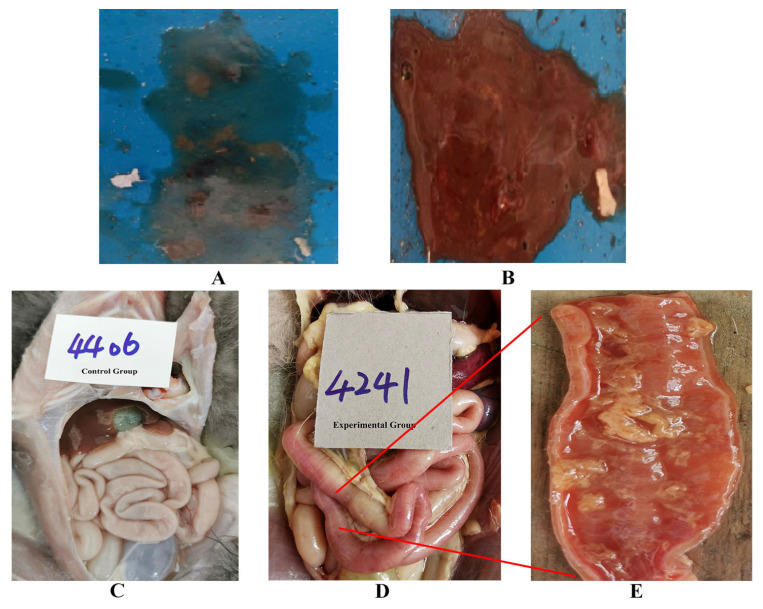
Typical symptoms of FPLV infection (diarrhoea, haematochezia, and intestinal bleeding): (**A**): diarrhoea; (**B**): haematochezia; (**C**): normal intestinal tract of cats; (**D**): intestinal bleeding in cats; (**E**): local anatomy of the bleeding intestinal tract.

**Figure 5:**
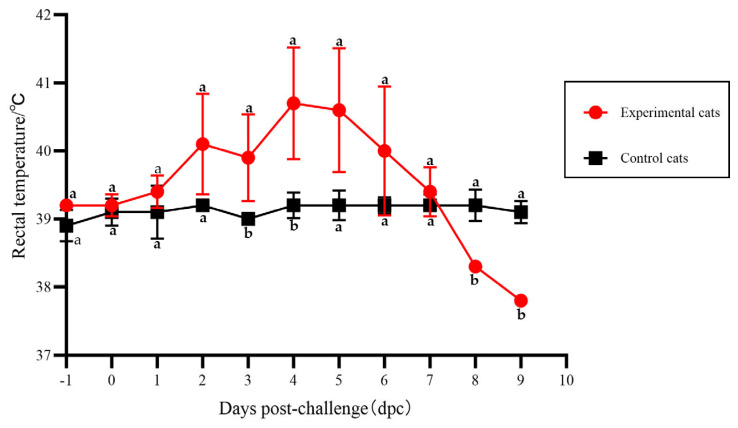
The changing curve of body temperature in cats from the different groups. Note: The different lowercase letters indicate a significant difference at the 0.05 level.

The pathogenicity of the Ala91Ser FPLV variant was evaluated in this study. After four days of infection, some cats in the experimental group displayed the typical symptoms of FPLV infection, such as depression, loss of appetite, vomiting, diarrhoea (Figure 4A), haematochezia (Figure 4B), leukopenia (Table 4), and high fever (Figure 5).

**Figure 6:**
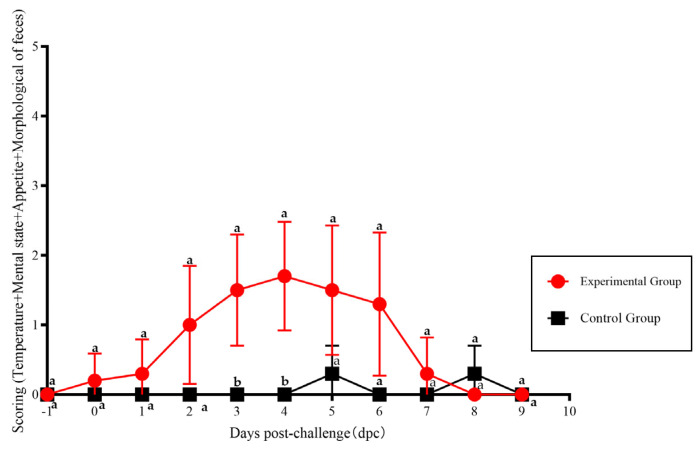
The scoring of clinical symptoms of FPLV-CC19-02 infection. Note: The different lowercase letters indicate a significant difference at the 0.05 level.

The scores calculated from the clinical symptoms of the various experimental groups are shown in Figure 6. The scores of cats in the experimental group exhibit a significant difference compared with those of the control group at 4–5 dpi (*p* < 0.05).

**Figure 7:**
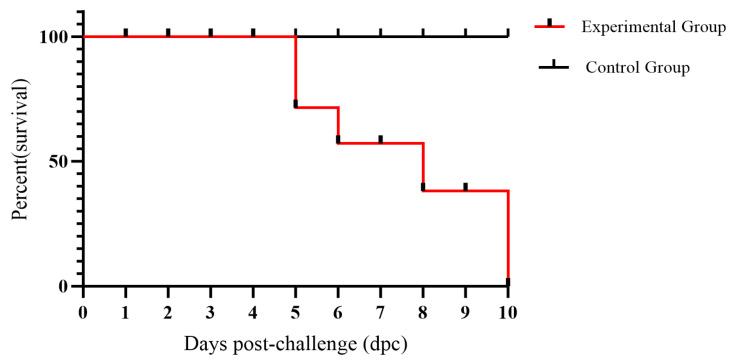
The survival chart of cats in the experimental and control groups.

Cats with severe infections exhibited absolute anorexia, dehydration, weight loss and leukopenia, and ultimately died. Two of the six cats in the experimental group died at 5 days post-infection (dpi), while another three cats died at 6, 8, and 9 dpi, respectively. The last cat in the experimental group perished at 11 dpi during the observation period of the experiment (Figure 7).

The body temperatures of some cats reached the scoring standard of 39.5 °C at 2 dpi and then continued to rise to a maximum temperature of 41.5 °C. As the disease progressed, the body temperatures of some cats decreased to levels below normal. The body temperature of cats in the experimental group shows a significant difference compared with cats in the control group at 4–5 dpi (*p* < 0.05) (Figure 5).

The haematological examination results showed that the WBC values of the affected cats displayed a downward trend, with some cats having values lower than 5 × 10^9^ cells/L at three days post-infection (dpi), and the values from some samples even decreasing to 0.63 × 10^9^ cells/L before the cats died (Table 4).

Virus shedding in the faeces following infection was detected on site using a rapid immunochromatographic strip, and parts of the results are displayed in Figure S1 in the Supplementary Materials. There was no virus shedding detected in the faeces of any cat on the first day after inoculation. Two-thirds (4 of 6) of the cats began shedding the virus in their faeces at 3 dpi. By four dpi, 5 of the 6 cats were shedding the virus in their faeces. Interestingly, one cat was found to be shedding virus in its faeces only at 3 dpi and was not detected as shedding the virus in its faeces at 4 and 6 dpi.

**Figure 8:**
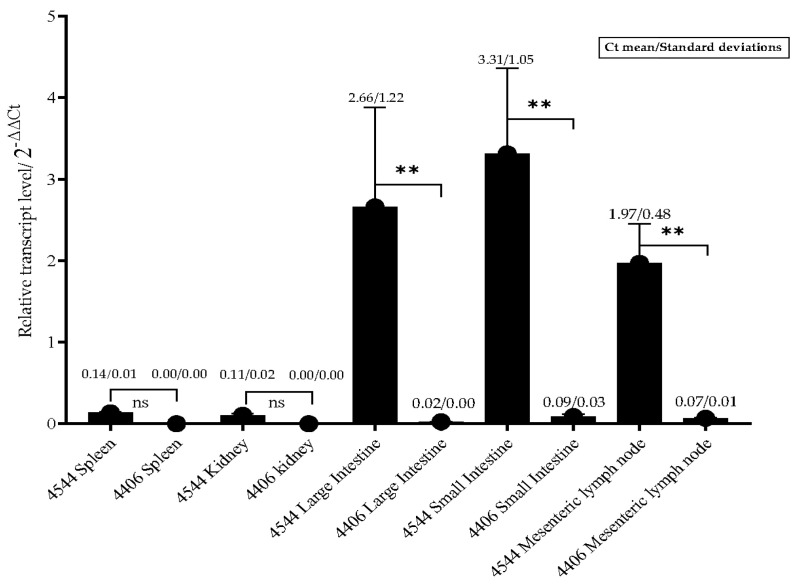
The relative transcript level of FPLV in different organs from cats in other groups. Notes: “ns” indicates no difference; “**” indicates significant difference at 0.01 level.

The distribution of FPLV in the various tissue organs of cats (from the experimental or control group) is illustrated in Figure 8. FPLV predominantly colonises the intestinal tract and mesenteric lymph nodes, and a significant difference was observed between the experimental and control groups at 4–5 dpi (ID4544 vs. ID4406, *p* < 0.01). The viral load in the spleen and kidney was low, with no significant difference noted between the experimental and control groups (ID4544 vs. ID4406). In the control group, the viral load in all organs was nearly undetectable.

### 3.5. Immunogenicity Study of the Ala91S FPLV Variant in the Cat

**Figure 9:**
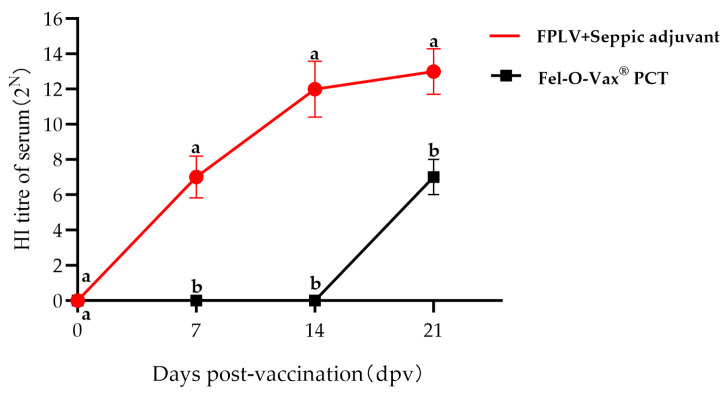
Results of the hemagglutination inhibition (HI) antibody titres of all cats after vaccination. Note: The different lowercase letters indicate a significant difference at the 0.05 level.

The immunogenicity of the Ala91Ser FPLV variant was also examined in this study. Seven days post-vaccination (dpv), two cats in Group I (receiving killed FPLV vaccine) exhibited haemagglutination inhibition (HI) antibodies (red line/scatter plot). All cats in this group showed higher levels of HI antibodies at 14 dpv. Furthermore, all cats vaccinated with either the killed vaccine or the commercial vaccine exhibited increased HI antibody levels at 21 days post-vaccination (dpv). The HI antibody titres of cats in Group I were significantly higher than those in Group II at 7, 14, and 21 dpv (*p* < 0.05) (Figure 9).

## 4. Discussion

The Ala91Ser FPLV variant has become widespread among cats in China since 2017, with no apparent reason for its re-emergence. This poses a significant threat to the growing Chinese pet industry, as even some vaccinated cats have succumbed to the virus [25]. Furthermore, the variant has frequently been identified in FPLVs circulating in Vietnam [26] and India [27], suggesting that it is beginning to prevail in Southeast Asia. Therefore, more attention should be given to this issue.

In this study, we isolated an epidemic Ala91Ser FPLV strain (named FPLV-CC19-02) from a clinical faecal sample. Genetic analysis revealed that the Ala91Ser FPLV strain displays a significant genetic divergence from the all commercially available pet vaccine strains such as M38246.1 (Fel-O-Vax^®^ PCT, Zoetis Inc., New York, NY, USA), EU498680.1 (PUREVAX^®^ Feline 3, Boehringer Ingelheim Inc., Ingelheim am Rhein, Rheinland-Pfalz, Germany), EU498681.1 (Felocell3^®^, Pfizer Inc., New York, NY, USA), OQ615264.1 (Nobivac^®^ Feline 1-HCP, MERCK Aimal Health USA Inc., Boxmeer, The Netherlands), and D88287.1 (Purevax^®^ RCP, Merial Inc., Paris, France), as illustrated in the phylogenetic trees (Figure 2). Notably, compared to the prototype FPLV strain Cu-4 (M38246.1), which serves as the basis for the only feline vaccine approved for use in China, the Ala91Ser strain exhibits amino acid substitutions at positions 91 (alanine to serine) and 101 (isoleucine to threonine) within the VP2 protein. These mutations were speculated to change the conformation of loops of the VP2 protein, further affect the receptor-binding ability of the VP2 protein, and, finally, influence the antigenicity of FPLV [28,29].

Furthermore, this research has demonstrated that the isolated FPLV variant can produce severe clinical signs of parvovirus infection in cats, such as a high fever, haematochezia, leukopenia, and a high mortality rate (100%), indicating that FPLV-CC19-02 is a virulent strain. There is no specific therapy for feline panleukopenia. Prevention is vital to your cat’s health, and it all starts with vaccination. Several FPLV vaccines have been approved for the prophylaxis of feline panleukopenia globally, and Fel-O-Vax^®^ PCT commands significant market shares, especially in China (almost 100%). Given this situation, numerous Chinese scientific research institutions have initiated the development of FPLV vaccines, aiming to expedite the prevention and control of feline panleukopenia in domestic cats.

Collectively, the findings in this study may provide valuable data for the development of a locally produced pet cat vaccine in China and may offer assistance in immune prophylaxis against FPL. However, much more research work is required before developing an effective vaccine, like preparing and safely storing a pre-master seed and subsequently a GMP master seed of the chosen production antigen, followed by quality (manufacture), safety, and efficacy tests, field trials, and so on. The discovery of the Ala91Ser FPLV strain also underscores the importance of ongoing virological surveillance and research in safeguarding feline health globally.

## 5. Conclusions

In this study, a Chinese feline panleukopenia virus epidemic strain, named FPLV-CC19-02, was successfully isolated from clinical faecal samples. This harboured typical mutations at amino acid positions 91 (Ala91Ser) and 101 (Ile101Thr) in the VP2 protein and exhibited a distant relationship with all commercial vaccine strains. The strain shows high virulence in cats and can induce cats to produce high levels of HI (hemagglutination inhibition) antibodies. Our results provide valuable data for the development of a Chinese-locally produced pet cat vaccine and can offer assistance in immune prophylaxis against FPLV.

## Figures and Tables

**Table 1:** Clinical symptoms and grading criteria of feline panleukopenia.

	Scores			
	0	1	2	3
Rectal temperature/°C	38.5–39.4 °C	39.5–39.9 °C	40–40.5 °C	>40.5
Mental state	Normal	Depression or lethargy	Unconscious	Dead
Appetite	Normal	Anorexia	Loss of appetite	—
Vomiting	Normal	Less	Much	—
Faeces	Normal	Diarrhoea	Haematochezia	—

**Table 2:** Nonsynonymous mutation analysis of the VP2 gene in different FPLV strains.

The Mutation Site in the Protein Sequence of VP2		91		101		232		562
The Mutation Site in the Nucleotide Sequence of VP2	271		302		694		1648	
PLI-IV/Vibac Vaccine/Japan/1996/D88287.1	G	Ala	C	Thr	A	Ile	C	Leu
Cu-4/Zoetis vaccine/USA/1991/M38246.1	G		T	Ile	G	Val	G	Val
Felocell/Pfizer vaccine/Italy/2008/EU498681.1	G		C	Thr	A	Ile	C	Leu
Nobivac/MSD vaccine/Brazil/2023/OQ615264.1	G		T	Ile	A		C	
Purevax/Merial vaccine/Italy/2008/EU498680.1	G		T		A		C	
CC19-02/Jilin/China/2019/OP471917.1	T	Ser	C	Thr	G	Val	G	Val
KF002/South Korea/2007/EU252146.1	T		C		G		G	
K49/South Korea/2008/HQ184195.1	T		C		G		G	
42/06-G3/Italy/2006/EU498699.1	T		C		G		G	

**Table 3:** Synonymous mutation analysis of the VP2 gene in different FPLV strains.

The Mutation Site in the VP2 Protein Sequence		57		100		221		270		291		347		507		524
The Mutation Site in the VP2 Nucleotide Sequence	171		300		663		810		871		1041		1521		1572	
PLI-IV/Vibac Vaccine /Jpan/1996/D88287.1	A	Gly	C	Asp	T	Ser	T	Cys	C	Leu	G	Ala	G	Thr	T	Tyr
Cu-4/Zoetis vaccine /USA/1991/M38246.1	G		T		T		C		T		G		G		T	
Felocell/Pfizer vaccine /Ialy/2008/EU498681.1	A		T		T		T		C		G		G		T	
Nobivac/MSD vaccine /Brzil/2023/OQ615264.1	A		C		T		T		C		G		A		C	
Purevax/Merial vaccine /Italy/2008/EU498680.1	A		C		T		T		C		G		G		T	
CC19-02/Jilin/China /2019/OP471917.1	G		T		C		T		T		A		A		C	
KF002/South Korea /2007/EU252146.1	G		T		T		T		T		A		A		C	
K49/South Korea/2008 /HQ184195.1	G		T		T		T		T		A		A		C	
42/06-G3/Italy/2006 /EU498699.1	G		T		T		T		C		A		A		C	

**Table 4:** The white blood cell counts of the peripheral blood samples of cats (10^9^ cells/L).

Group	Number	Electronic Tag	Days Post Inoculation (dpi)				Reference Range
			1	3	5	7	
Experiment group	1	4258	15.74	10.61	8.94	5.72	5.50–19.50
	2	4260	14.21	10.86	—	—	
	3	4402	12.98	4.73	0.82	—	
	4	4544	13.63	9.32	4.62	0.91	
	5	4243	12.2	6.73	2.93	0.63	
	6	4241	17.2	2.87	—	—	
Control group	1	4254	13.15	13.64	14.25	12.5	
	2	4257	16.57	15.62	15.26	16.1	
	3	4406	14.46	13.32	14.5	15.62	

Note: “—” indicates that the cat died.

## Data Availability

All data are contained within this manuscript and Supplementary Materials.

## Supplementary Materials

The following supporting information may be downloaded at: https://www.mdpi.com/article/10.3390/vetsci12070668/s1. Table S1: Reference FPLV strain used in constructing the phylogenetic tree; Table S2: Synonymous mutation analysis between FPLV-CC19-02 and typical FPLV strains; Table S3: Nonsynonymous mutation analysis between FPLV-CC19-02 and typical FPLV strains; Table S4: Screening for Ala91Ser FPLV variants in the NCBI database. Figure S1: Immunochromatographic strip detection of FPLV shedding in faeces.

## Abbreviations

The following abbreviations are used in this manuscript:

FPL	Feline panleukopenia
FPLV	Feline panleukopenia virus
dpv	Days post-vaccination
dpi	Days post-inoculation
