# Supplementary material for: Genetic and Biological Properties of an Epidemic Feline Panleukopenia Virus Strain (Ala91Ser) in China
Source: Vet Sci. 2025 Jul 16;12(7):668. doi: 10.3390/vetsci12070668 (PMC12300517; doi:10.3390/vetsci12070668)
Supplement: Supplementary file 1 [file vetsci-12-00668-s001.zip › vetsci-3716090-supplementary.pdf]

Supplementary material

Table S1 Reference FPLV strain used in construct Phylogenetic tree

No.	Name of FPLV strains	Per. Ident (%)	Accession
1	CC-19-02	100	OR921195.1
2	QD22-13	100	OR727319.1
3	QD22-8	100	OR727317.1
4	YZ2019-026	100	OQ863618.1
5	YZ2019-025	100	OQ863617.1
6	YZ2018-023	100	OQ863615.1
7	Panthera leo Parvovirus Z30	100	OP745049.1
8	F-D33	100	OL547736.1
9	F-D34	100	OL547735.1
10	F-D	100	OL547732.1
11	FPV-SH2002	100	MW659466.1
12	C-DY6	100	OK128324.1
13	FPV-SH2003	100	MW811187.1
14	SH2001	100	MW650831.1
15	CPVTS27	100	MT179777.1

Continued from the above table

No.	Name of FPLV strains	Per. ident	Accession
1	Beijing-01/2018	99.94	MK266797.1
2	cat-6	99.94	PP738175.1
3	cat-2	99.94	PP738171.1
4	40	99.94	OQ398400.1
5	QD22-3	99.94	OR727318.1
6	QD22-4	99.94	OR727316.1
7	Xinxiang-05	99.94	OR551226.1
8	Luoyang-08	99.94	OR551219.1
9	AHWH52	99.94	OR211675.1
10	HN2101	99.94	OQ868566.1
11	AH2012	99.94	OQ868564.1
12	AH2002	99.94	OQ868555.1
13	HB2001	99.94	OQ868548.1
14	HN1910	99.94	OQ868547.1
15	HN1904	99.94	OQ868542.1
16	HN1803	99.94	OQ868535.1
17	FJFZ2	99.94	OR194134.1
18	FJFZ1	99.94	OR194133.1
19	GDGZ4	99.94	OR194132.1
20	HNZZ1	99.94	OR194126.1

21	Yanji33	99.94	OM918779.1
22	Yanji25	99.94	OM918772.1
23	Yanji24	99.94	OM918771.1
24	Yanji18	99.94	OM885384.1
25	Yanji4	99.94	OM885374.1
26	F-A	99.94	OL547737.1
27	F-E	99.94	OL547734.1
28	SD/2019/4	99.94	OK384312.1
29	SD/2019/2	99.94	OK384310.1
30	SMU-D3	99.94	MZ442312.1
31	ZJFPV11	99.94	MW495836.1
32	ZJFPV6	99.94	MW495832.1
33	ZJHN-135	99.94	MW017616.1
34	BJ090	99.94	MT270576.1
35	BJ094	99.94	MT270575.1
36	BJ308	99.94	MT270569.1
37	BJ338	99.94	MT270565.1
38	BJ624	99.94	MT270540.1
39	BJ663	99.94	MT270534.1
40	BJ728	99.94	MT270531.1
41	DLC15	99.94	MN419012.1
42	DLC03	99.94	MN418999.1
43	HF1	99.94	MT614366.1
44	KF002	99.94	EU252146.1
45	30	99.94	OQ398395.1

Continued from the above table

No.	Name of FPLV strains	Per. ident	Accession
1	QDDX	99.89	MK301396.1
2	cat-3	99.89	PP738172.1
3	FPV085	99.89	OQ398412.1
4	FPV082	99.89	OQ398411.1
5	Zhengzhou-01	99.89	OR551222.1
6	77	99.89	OR399567.1
7	42	99.89	OR399563.1
8	JS2201	99.89	OQ868568.1
9	JS2002	99.89	OQ868565.1
10	AH2010	99.89	OQ868563.1
11	AH2001	99.89	OQ868554.1
12	AH1903	99.89	OQ868552.1
13	HB2002	99.89	OQ868549.1
14	HN1804	99.89	OQ868536.1

15	GDGZ2	99.89	OR194130.1
16	GDGZ1	99.89	OR194129.1
17	HNZZ3	99.89	OR194128.1
18	SH5	99.89	OR194125.1
19	SH2	99.89	OR194122.1
20	SDQD21	99.89	OQ535504.1
21	Yanji26	99.89	OM918784.1
22	Yanji17	99.89	OM918783.1
23	Yanji31	99.89	OM918777.1
24	Yanji13	99.89	OM885382.1
25	Yanji10	99.89	OM885379.1
26	F-D35	99.89	OL547733.1
27	SD/2019/6	99.89	OK384314.1
28	SD/2019/5	99.89	OK384313.1
29	SD/2019/3	99.89	OK384311.1
30	ZJFPV14	99.89	MW495839.1
31	ZJFPV2	99.89	MW495829.1
32	K49	99.89	HQ184195.1
33	42/06-G3	99.89	EU498699.1
34	KF003	99.89	EU252147.1

Continued from the above table

1	Name of FPLV strains	Per. ident	Accession
2	S2	99.83	OQ815875.1
3	LZ05	99.83	OQ869254.1
4	JS2001	99.83	OQ868562.1
5	AH2003	99.83	OQ868556.1
6	AH1904	99.83	OQ868553.1
7	AH1902	99.83	OQ868551.1

Table S2 Synonymous mutation analysis between FPLV-CC19-02 and typical FPLV strains

	Synonymous mutation				Synonymous mutation			
	Mutation site of nucleotide sequence			Mutation site of Protein sequence	Mutation site of nucleotide sequence			Mutation site of Protein sequence
	**169**	**170**	**171**	P57	**298**	**299**	**300**	P100
PLI-IV/FPLV/Vibac Vaccine/Japan/1996/D88287.1	G	G	A	Gly(G)	G	A	C	Asp(D)
Cu-4/FPLV/Zoetis vaccine/USA/1991/M38246.1	G	G	G	Gly(G)	G	A	T	Asp(D)
Felocell/FPLV/Pfizer vaccine/Italy/2008/EU498681.1	G	G	A	Gly(G)	G	A	T	Asp(D)
Nobivac/FPLV/MSD vaccine/Brazil/2023/OQ615264.1	G	G	A	Gly(G)	G	A	C	Asp(D)
Purevax/Merial vaccine/Italy/2008/EU498680.1	G	G	A	Gly(G)	G	A	T	Asp(D)
CC19-02/FPLV/Jilin/China/2019/OP471917.1	G	G	G	Gly(G)	G	A	T	Asp(D)
KF002/South Korea/2007/EU252146.1	G	G	G	Gly(G)	G	A	T	Asp(D)
K49/South Korea/2008/HQ184195.1	G	G	G	Gly(G)	G	A	T	Asp(D)
42/06-G3/Italy/2006/EU498699.1	G	G	G	Gly(G)	G	A	T	Asp(D)
	**748**	**749**	**750**	**P250**	**808**	**809**	**810**	**P270**
PLI-IV/FPLV/Vibac Vaccine/Japan/1996/D88287.1	G	T	G	Val(V)	T	G	T	Cys(C)
Cu-4/FPLV/Zoetis vaccine/USA/1991/M38246.1	G	T	G	Val(V)	T	G	C	Cys(C)
Felocell/FPLV/Pfizer vaccine/Italy/2008/EU498681.1	G	T	G	Val(V)	T	G	T	Cys(C)
Nobivac/FPLV/MSD vaccine/Brazil/2023/OQ615264.1	G	T	G	Val(V)	T	G	T	Cys(C)
Purevax/Merial vaccine/Italy/2008/EU498680.1	G	T	G	Val(V)	T	G	T	Cys(C)
CC19-02/FPLV/Jilin/China/2019/OP471917.1	G	T	A	Val(V)	T	G	T	Cys(C)
KF002/South Korea/2007/EU252146.1	G	T	A	Val(V)	T	G	T	Cys(C)
K49/South Korea/2008/HQ184195.1	G	T	A	Val(V)	T	G	T	Cys(C)
42/06-G3/Italy/2006/EU498699.1	G	T	A	Val(V)	T	G	T	Cys(C)

Continued from the above table								
	**871**	**872**	**873**	**P291**	**1039**	**1040**	**1041**	**P347**
PLI-IV/FPLV/Vibac Vaccine/Japan/1996/D88287.1	C	T	A	Leu(L)	G	C	G	Ala(A)
Cu-4/FPLV/Zoetis vaccine/USA/1991/M38246.1	T	T	A	Leu(L)	G	C	G	Ala(A)
Felocell/FPLV/Pfizer vaccine/Italy/2008/EU498681.1	C	T	A	Leu(L)	G	C	G	Ala(A)
Nobivac/FPLV/MSD vaccine/Brazil/2023/OQ615264.1	C	T	A	Leu(L)	G	C	G	Ala(A)
Purevax/Merial vaccine/Italy/2008/EU498680.1	C	T	A	Leu(L)	G	C	G	Ala(A)
CC19-02/FPLV/Jilin/China/2019/OP471917.1	T	T	A	Leu(L)	G	C	A	Ala(A)
KF002/South Korea/2007/EU252146.1	T	T	A	Leu(L)	G	C	A	Ala(A)
K49/South Korea/2008/HQ184195.1	T	T	A	Leu(L)	G	C	A	Ala(A)
42/06-G3/Italy/2006/EU498699.1	C	T	A	Leu(L)	G	C	A	Ala(A)
	**1519**	**1520**	**1521**	**P507**	**1570**	**1571**	**1572**	**P524**
PLI-IV/FPLV/Vibac Vaccine/Japan/1996/D88287.1	A	C	G	Thr(T)	T	A	T	Tyr(Y)
Cu-4/FPLV/Zoetis vaccine/USA/1991/M38246.1	A	C	G	Thr(T)	T	A	T	Tyr(Y)
Felocell/FPLV/Pfizer vaccine/Italy/2008/EU498681.1	A	C	G	Thr(T)	T	A	T	Tyr(Y)
Nobivac/FPLV/MSD vaccine/Brazil/2023/OQ615264.1	A	C	A	Thr(T)	T	A	C	Tyr(Y)
Purevax/Merial vaccine/Italy/2008/EU498680.1	A	C	G	Thr(T)	T	A	T	Tyr(Y)
CC19-02/FPLV/Jilin/China/2019/OP471917.1	A	C	A	Thr(T)	T	A	C	Tyr(Y)
KF002/South Korea/2007/EU252146.1	A	C	A	Thr(T)	T	A	C	Tyr(Y)
K49/South Korea/2008/HQ184195.1	A	C	A	Thr(T)	T	A	C	Tyr(Y)
42/06-G3/Italy/2006/EU498699.1	A	C	A	Thr(T)				

Table S3 Non-synonymous mutation analysis between FPLV-CC19-02 and typical FPLV strains

	Non-synonymous mutation				Non-synonymous mutation			
	Mutation at site of nucleotide sequence			Mutation at site of Protein sequence	Mutation at site of nucleotide sequence			Mutation at site of Protein sequence
	**271**	**272**	**273**	**P91**	**301**	**302**	**303**	P101
PLI-IV/FPLV/Vibac Vaccine/Japan/1996/D88287.1	G	C	A	Ala(A)	A	C	T	Thr (T)
Cu-4/FPLV/Zoetis vaccine/USA/1991/M38246.1	G	C	A	Ala(A)	A	T	T	Ile (I)
Felocell/FPLV/Pfizer vaccine/Italy/2008/EU498681.1	G	C	A	Ala(A)	A	C	T	Thr (T)
Nobivac/FPLV/MSD vaccine/Brazil/2023/OQ615264.1	G	C	A	Ala(A)	A	C	T	Thr (T)
Purevax/Merial vaccine/Italy/2008/EU498680.1	G	C	A	Ala(A)	A	T	T	Ile (I)
CC19-02/FPLV/Jilin/China/2019/OP471917.1	T	C	A	Ser (S)	A	C	T	Thr (T)
KF002/South Korea/2007/EU252146.1	T	C	A	Ser (S)	A	C	T	Thr (T)
K49/South Korea/2008/HQ184195.1	T	C	A	Ser (S)	A	C	T	Thr (T)
42/06-G3/Italy/2006/EU498699.1	T	C	A	Ser (S)	A	C	T	Thr (T)
	**694**	**695**	**696**	**P232**	**1684**	**1685**	**1686**	**P562**
PLI-IV/FPLV/Vibac Vaccine/Japan/1996/D88287.1	A	T	A	Ile(I)	C	T	A	Leu(L)
Cu-4/FPLV/Zoetis vaccine/USA/1991/M38246.1	G	T	A	Val(V)	G	T	A	Val(V)
Felocell/FPLV/Pfizer vaccine/Italy/2008/EU498681.1	A	T	A	Ile(I)	C	T	A	Leu(L)
Nobivac/FPLV/MSD vaccine/Brazil/2023/OQ615264.1	A	T	A	Ile(I)	C	T	A	Leu(L)
Purevax/Merial vaccine/Italy/2008/EU498680.1	A	T	A	Ile(I)	C	T	A	Leu(L)
CC19-02/FPLV/Jilin/China/2019/OP471917.1	G	T	A	Val(V)	G	T	A	Val(V)
KF002/South Korea/2007/EU252146.1	G	T	A	Val(V)	G	T	A	Val(V)
K49/South Korea/2008/HQ184195.1	G	T	A	Val(V)	G	T	A	Val(V)
42/06-G3/Italy/2006/EU498699.1	G	T	A	Val(V)	G	T	A	Val(V)

Table S4-Screening Ala91Ser FPLV variants in NCBI database

No.	Gene ID	Name of strains	Host	Country	Year	Site 91	Proportion of variant
1	D88287	PLI-IV	Felis catus	Japan	1968	A	0/1
2	AB000056	Obihiro	Felis catus	Japan	1974	A	0/1
3	AB000068	TU4	Felis catus	Japan	1975	A	0/5
4	D78584	TU10	Felis catus	Japan	1975	A	
5	AB000064	TU12	Felis catus	Japan	1975	A	
6	AB000066	TU2	Felis catus	Japan	1975	A	
7	AB000070	TU8	Felis catus	Japan	1975	A	
8	JN867595	TX/Rac2.2/78	Procyon lotor	USA	1978	A	0/3
9	KM624023	TX/Rac3/1978	Procyon lotor	USA	1978	A	
10	JN867596	TXRac1.2/78	Procyon lotor	USA	1978	A	
11	D88286	483	Felis catus	Japan	1990	A	0/2
12	JN867594	NJ/RPV-6/90	Procyon lotor	USA	1990	A	
13	AB000054	Fukagawa	Felis catus	Japan	1993	A	0/1
14	AB000050	94-1	Felis catus	Japan	1994	A	0/3
15	AB000052	AO1	Felis catus	Japan	1994	A	
16	AB000059	Som1	Felis catus	Japan	1994	A	
17	AB000061	Som4	Felis catus	Japan	1995	A	0/1
18	AF015223	T1	leopard cat	China/Taiwan	1996	A	0/1
19	AB054225	V142	Felis catus	Vietname	1997	A	0/3
20	AB054226	V208	Felis catus	Vietname	1997	A	
21	AB054227	V211	Felis catus	Vietname	1997	A	
22	EU498682	198/01	Felis catus	Italy	2001	A	0/1
23	EU498684	103/02	Felis catus	Italy	2002	A	0/2
24	EU498683	41/02	Felis catus	Italy	2002	A	
25	EU498685	150/03	Felis catus	Italy	2003	A	0/3
26	EU498686	189/03	Felis catus	Italy	2003	A	
27	EU498687	30/003	Felis catus	Italy	2003	A	
28	AB262659	SL1	Snow leopard	Japan	2004	A	1/12
29	EU498688	134/04-1	Felis catus	Italy	2004	A	

30	EU498689	134/04-2	Felis catus	Italy	2004	A	
31	EU498690	134/04-3	Felis catus	Italy	2004	A	
32	EU498691	134/04-5	Felis catus	Italy	2004	A	
33	EU498692	143/04	Felis catus	Italy	2004	A	
34	EU498693	355/04	Felis catus	Italy	2004	A	
35	DQ474237	GT-3	Tiger	China/Guangxi	2004	A	
36	DQ474235	HT-69	Tiger	China/Heilongjiang	2004	A	
37	DQ474236	JF-1	Tiger	China/Jilin	2004	A	
38	DQ099431	JF-3	Tiger	China/Jilin	2004	S	
39	DQ474238	SM-4	Tiger	China/Shaanxi	2004	A	
40	DQ099430	ZF-5	Tiger	China/Henan	2004	A	
41	EU498695	119/05	Felis catus	Italy	2005	A	0/2
42	EU498694	20/05	Felis catus	Italy	2005	A	
43	EU498713	97/06-10	Felis catus	Italy	2006	A	2/27 (7%)
44	EU498714	97/06-11	Felis catus	Italy	2006	A	
45	EU498696	22/06	Felis catus	Italy	2006	A	
46	EU498715	228/06	Felis catus	Italy	2006	A	
47	EU498707	42/06-12	Felis catus	Italy	2006	A	
48	EU498710	42/06-17	Felis catus	Italy	2006	A	
49	EU498711	42/06-18	Felis catus	Italy	2006	A	
50	EU498697	42/06-G1	Felis catus	Italy	2006	A	
51	EU498705	42/06-G10	Felis catus	Italy	2006	A	
52	EU498706	42/06-G11	Felis catus	Italy	2006	S	
53	EU498708	42/06-G14	Felis catus	Italy	2006	A	
54	EU498709	42/06-G16	Felis catus	Italy	2006	A	
55	EU498712	42/06-G19	Felis catus	Italy	2006	A	
56	EU498698	42/06-G2	Felis catus	Italy	2006	A	
57	EU498699	42/06-G3	Felis catus	Italy	2006	S	
58	EU498700	42/06-G4	Felis catus	Italy	2006	A	
59	EU498701	42/06-G5	Felis catus	Italy	2006	A	
60	EU498702	42/06-G6	Felis catus	Italy	2006	A	

61	EU498703	42/06-G7	Felis catus	Italy	2006	A	
62	EU498704	42/06-G8	Felis catus	Italy	2006	A	
63	KU248456	PT001/06	Felis catus	Portugal	2006	A	
64	KU248457	PT002/06	Felis catus	Portugal	2006	A	
65	KU248458	PT003/06	Felis catus	Portugal	2006	A	
66	KU248459	PT004/06	Felis catus	Portugal	2006	A	
67	KU248460	PT005/06	Felis catus	Portugal	2006	A	
68	KU248461	PT006/06	Felis catus	Portugal	2006	A	
69	KT240128	PT020/06	Felis catus	Portugal	2006	A	
70	EU697383	HT-262	Tiger	China/Heilongjiang	2007	A	4/17 (24%)
71	EU360959	1335/07	Felis catus	Hungary	2007	S	
72	EU145593	389/07	Asian palm civet	Hungary	2007	A	
73	EU498718	443/07	Felis catus	Italy	2007	A	
74	EU498719	490/07	Felis catus	Italy	2007	A	
75	EU498720	498/07	Felis catus	Italy	2007	A	
76	EU498716	50/07-1	Felis catus	Italy	2007	A	
77	EU498717	50/07-2	Felis catus	Italy	2007	A	
78	EU360958	933/07	Felis catus	Hungary	2007	S	
79	EU697387	HT-163	Tiger	China/Heilongjiang	2007	A	
80	EU697384	HT-290	Tiger	China/Heilongjiang	2007	A	
81	EU697386	HT-374	Tiger	China/Heilongjiang	2007	A	
82	EU252145	KF001	Felis catus	South Korea	2007	A	
83	EU252146	KF002	Felis catus	South Korea	2007	S	
84	EU252147	KF003	Felis catus	South Korea	2007	S	
85	KT240129	PT001/07	Felis catus	Portugal	2007	A	
86	KU248462	PT002/07	Felis catus	Portugal	2007	A	
87	GQ857595	BFPV	Blue fox	China/Shandong	2008	A	2/26 (8%)
88	OR566997	RJ9222008	Felis catus	Brazil	2008	S	

89	FJ231389	BJ-22	Monkey	China/Beijing	2008	A	
90	FJ936171	ChangC2007	Felis catus	China/Jilin	2008	A	
91	EU498681	Felocell-Pfizer vaccine	Felis catus	Italy	2008	A	
92	FJ405225	FPV	Tiger	China/Jilin	2008	A	
93	HQ184189	K2	Felis catus	South Korea	2008	A	
94	HQ184193	K22	Felis catus	South Korea	2008	A	
95	HQ184194	K23	Felis catus	South Korea	2008	A	
96	HQ184190	K3	Felis catus	South Korea	2008	A	
97	HQ184191	K49	Felis catus	South Korea	2008	A	
98	HQ184195	K49	Felis catus	South Korea	2008	S	
99	HQ184196	K50	Felis catus	South Korea	2008	A	
100	HQ184192	K7	Felis catus	South Korea	2008	A	
101	HQ184197	KS11	Felis catus	South Korea	2008	A	
102	HQ184198	KS18	Felis catus	South Korea	2008	A	
103	HQ184204	KS2	Felis catus	South Korea	2008	A	
104	HQ184199	KS23	Felis catus	South Korea	2008	A	
105	HQ184200	KS42	Felis catus	South Korea	2008	A	
106	HQ184201	KS45	Felis catus	South Korea	2008	A	
107	HQ184202	KS47	Felis catus	South Korea	2008	A	
108	HQ184203	KS58	Felis catus	South Korea	2008	A	
109	KT240130	PT005/08	Felis catus	Portugal	2008	A	
110	KU248463	PT015/08	Felis catus	Portugal	2008	A	

111	KT240131	PT022/08	Felis catus	Portugal	2008	A	
112	EU498680	Purevax-Merial vaccine	Felis catus	Italy	2008	A	
113	JF422105	PT09	Egyptian mongoose	Portugal	2009	A	
114	JX475259	CO/545/10	Puma concolor	USA	2010	A	
115	JX475253	CO/546/10	Puma concolor	USA	2010	T	
116	JX475245	CO/952/10	Puma concolor	USA	2010	A	
117	JX475254	CO/977/10	Puma concolor	USA	2010	A	0/8
118	OR566998	RJ10182010	Felis catus	Brazil	2010	A	
119	JN867593	CA/208-A/10	Procyon lotor	USA	2010	A	
120	JX411926	PT10-newCPV-2b	Stone marten	Portugal	2010	A	
121	JX475256	CO/1103/11	Puma concolor	USA	2011	A	
122	OR567000	RJ10962011	Felis catus	Brazil	2011	A	0/4
123	JX048608	FPV-1	Felis catus	China/Taiwan	2011	A	
124	OR566999	RJ10852011	Felis catus	Brazil	2011	A	
125	JX475270	GA/1/12	Procyon lotor	USA	2012	A	
126	OR567001	RJ11592012	Felis catus	Brazil	2012	S	
127	OR567002	RJ11602012	Felis catus	Brazil	2012	S	3/8 (38%)
128	OR567003	RJ11612012	Felis catus	Brazil	2012	S	
129	KC473946	GD1209YGP	Felis catus	China/Guangzhou	2012	A	
130	KJ813895	MA/188	Procyon lotor	USA	2012	A	

131	KJ813894	MA/190	Raccoon	USA	2012	A	
132	KT240132	PT183/12	Felis catus	Portugal	2012	A	
133	KT240134	PT210/13	Felis catus	Portugal	2013	A	
134	OR567004	RJ11892013	Felis catus	Brazil	2013	A	
135	OR567005	RJ11982013	Felis catus	Brazil	2013	S	1/5 (20%)
136	KJ813893	Bobcat/ND/979	Lynx rufus	USA	2013	A	
137	KT240133	PT083/13	Felis catus	Portugal	2013	A	
138	OP985508	C1	Felis catus	Nigeria	2014	A	
139	OP985509	C2	Felis catus	Nigeria	2014	A	
140	OP985510	C3	Felis catus	Nigeria	2014	A	
141	OP985512	C10	Felis catus	Nigeria	2014	A	
142	OP985513	C14	Felis catus	Nigeria	2014	A	0/18
143	OP985514	C18	Felis catus	Nigeria	2014	A	
144	OP985515	C32	Felis catus	Nigeria	2014	A	
145	OP985516	C39	Felis catus	Nigeria	2014	A	
146	OP985511	C5	Felis catus	Nigeria	2014	A	

147	OP985517	C51	Felis catus	Nigeria	2014	A	
148	OP985518	C58	Felis catus	Nigeria	2014	A	
149	OP985519	C64	Felis catus	Nigeria	2014	A	
150	OP985520	C71	Felis catus	Nigeria	2014	A	
151	OP985521	C78	Felis catus	Nigeria	2014	A	
152	OP985522	C92	Felis catus	Nigeria	2014	A	
153	KT240135	PT264/14	Felis catus	Portugal	2014	A	
154	KU248464	PT265/14	Felis catus	Portugal	2014	A	
155	KT240136	PT271/14	Felis catus	Portugal	2014	A	
156	MH669800	VT01	Prionodon linsang	Thailand	2015	A	
157	MK671151	16CC1106	Felis catus	China/Jilin	2016	A	
158	MK671154	16JZ0601	Felis catus	China/Liaoning	2016	A	
159	MK671155	16SY0601	Felis catus	China/Liaoning	2016	A	0/16
160	MF541121	CC-02/16	Felis catus	China/Jilin	2016	A	
161	MH329286	F2016019	Felis catus	China/Henan	2016	A	
162	MK671150	16CC0806	Felis catus	China/Jilin	2016	A	

163	MK671152	16JL0804	Felis catus	China/Jilin	2016	A	
164	MK671153	16JL1205	Felis catus	China/Jilin	2016	A	
165	MK671156	16SY0711	Felis catus	China/Liaoning	2016	A	
166	MF541119	BC-02/16	Felis catus	China/Jilin	2016	A	
167	MF541120	BJ-03/16	Felis catus	China/Beijing	2016	A	
168	MF541122	HRB-01/16	Felis catus	China/Heilongjiang	2016	A	
169	MF541123	JL-01/17-03	Felis catus	China/Jilin	2016	A	
170	MF541124	JL-03/17-05	Felis catus	China/Jilin	2016	A	
171	MF541125	JL-04/16	Felis catus	China/Jilin	2016	A	
172	MF541140	SP-01/16	Felis catus	China/Jilin	2016	A	
173	MK671157	17BC0704	Felis catus	China/Jilin	2017	A	5/49 (10%)
174	MK671159	17CC0308	Felis catus	China/Jilin	2017	S	
175	MK671160	17DD0501	Felis catus	China/Liaoning	2017	A	
176	MK671168	17SY0302	Felis catus	China/Liaoning	2017	A	
177	MK671169	17SY0402	Felis catus	China/Liaoning	2017	S	
178	MT274377	51 FOX	Red fox	Italy	2017	T	

179	MK982094	CHJL-Siberian Tiger-01	Tiger	China/Jilin	2017	A	
180	MK266782	Haerbin-05	Felis catus	China/Heilongjiang	2017	A	
181	MF541129	JL-12/17-05	Felis catus	China/Jilin	2017	A	
182	MF541132	JL-24/17-05	Felis catus	China/Jilin	2017	A	
183	MF541134	JL-29/17-05	Felis catus	China/Jilin	2017	A	
184	MK295775	JL-3	Felis catus	China/Jilin	2017	A	
185	MZ442312	SMU-D3	Felis catus	China/Sichuan	2017	S	
186	MK671158	17BC0801	Felis catus	China/Jilin	2017	A	
187	OP153925	17D01	Felis catus	South Korea	2017	A	
188	OP153926	17D02	Felis catus	South Korea	2017	A	
189	MK671161	17DD0902	Felis catus	China/Liaoning	2017	A	
190	MK671162	17HRB0505	Felis catus	China/Heilongjiang	2017	A	
191	MK671163	17HRB1001	Felis catus	China/Heilongjiang	2017	A	
192	MK671164	17JL0704	Felis catus	China/Jilin	2017	S	
193	MK671165	17JLSY0701	Felis catus	China/Jilin	2017	A	
194	MK671166	17JLSY0901	Felis catus	China/Jilin	2017	A	

195	MK671167	17SP0503	Felis catus	China/Jilin	2017	A	
196	MK671170	17SY0503	Felis catus	China/Liaoning	2017	A	
197	MK671171	17SY0902	Felis catus	China/Liaoning	2017	A	
198	MK266791	Chengdu-01	Felis catus	China/Sichuan	2017	A	
199	MK266790	Chengdu-03	Felis catus	China/Sichuan	2017	A	
200	MK266788	Guiyang01	Felis catus	China/Guizhou	2017	A	
201	MK266789	Guiyang01	Felis catus	China/Guizhou	2017	A	
202	MK266784	Haerbin-13	Felis catus	China/Heilongjiang	2017	A	
203	MK357739	HN3	Canis lupus familiaris	Vietnam	2017	A	
204	MZ508523	17DIAPD55048/2	Felis catus	Italy	2017	S	
205	MF541126	JL-04/17-03	Felis catus	China/Jilin	2017	A	
206	MF541127	JL-07/17-05	Felis catus	China/Jilin	2017	A	
207	MF541128	JL-10/17-06	Felis catus	China/Jilin	2017	A	
208	MF541130	JL-19/17-06	Felis catus	China/Jilin	2017	A	
209	MF541131	JL-20/17-05	Felis catus	China/Jilin	2017	A	
210	MF541133	JL-28/17-05	Felis catus	China/Jilin	2017	A	

211	MF541135	JL-33/17-05	Felis catus	China/Jilin	2017	A	
212	MF541136	JL-34/17-05	Felis catus	China/Jilin	2017	A	
213	MF541137	JL-40/17-05	Felis catus	China/Jilin	2017	A	
214	MF541138	JL-47/17-05	Felis catus	China/Jilin	2017	A	
215	MF541139	JT-01/17-03	Felis catus	China/Jilin	2017	A	
216	MK266792	Shenyang-01	Felis catus	China/Liaoning	2017	A	
217	MK266798	Shenyang-05	Felis catus	China/Liaoning	2017	A	
218	MK266786	Shenyang-19	Felis catus	China/Liaoning	2017	A	
219	MK266787	Shenyang-41	Felis catus	China/Liaoning	2017	A	
220	MK266785	Shenyang-5	Felis catus	China/Liaoning	2017	A	
221	MZ442309	SMU-D4	Felis catus	China/Sichuan	2017	A	
222	MK671172	18BC0504	Felis catus	China/Jilin	2018	A	
223	MK671173	18CC0102	Felis catus	China/Jilin	2018	A	
224	MK671177	18HRB0102	Felis catus	China/Heilongjiang	2018	A	
225	MK671178	18HRB0801	Felis catus	China/Heilongjiang	2018	A	
226	MK671181	18JL0602	Felis catus	China/Jilin	2018	A	14/66 (21%)

227	MW091486	CD-2	Giant panda	China/Sichuan	2018	A
228	ON646201	DL01	Felis catus	China/Liaoning	2018	A
229	ON646202	DL02	Felis catus	China/Liaoning	2018	A
230	ON646203	DL03	Felis catus	China/Liaoning	2018	A
231	ON646204	DL04	Felis catus	China/Liaoning	2018	A
232	ON646205	DL05	Felis catus	China/Liaoning	2018	S
233	ON646206	DL06	Felis catus	China/Liaoning	2018	A
234	ON646207	DL07	Felis catus	China/Liaoning	2018	A
235	MK357738	HN39AA	Canis lupus familiaris	Viet Nam	2018	A
236	MK301396	QDDX	Felis catus	China/Shandong	2018	S
237	MZ442307	SMU-D46	Felis catus	China/Sichuan	2018	A
238	MZ442306	SMU-D50	Felis catus	China/Sichuan	2018	A
239	MK671174	18CC0718	Felis catus	China/Jilin	2018	A
240	MK671175	18CC0909	Felis catus	China/Jilin	2018	S
241	OP153927	18D01	Felis catus	South Korea	2018	A
242	MK671176	18DD0302	Felis catus	China/Liaoning	2018	A

243	MK671179	18HRB1002	Felis catus	China/Heilongjiang	2018	S
244	MK671180	18JL0105	Felis catus	China/Jilin	2018	A
245	MK671182	18JZ0501	Felis catus	China/Liaoning	2018	A
246	MK671183	18LY0701	Felis catus	China/Liaoning	2018	A
247	MK671184	18LY0801	Felis catus	China/Liaoning	2018	S
248	MK671185	18LY0902	Felis catus	China/Liaoning	2018	S
249	MK671186	18QQHE0503	Felis catus	China/Heilongjiang	2018	A
250	MK671187	18SP0701	Felis catus	China/Jilin	2018	A
251	MK671188	18SY0102	Felis catus	China/Liaoning	2018	A
252	MK266797	Beijing-01	Felis catus	China/Beijing	2018	S
253	MK266795	Beijing-L3	Felis catus	China/Beijing	2018	A
254	MK266796	Beijing-L4	Felis catus	China/Beijing	2018	A
255	MZ322607	CD2018	Giant panda	China/Sichuan	2018	A
256	MT857283	F1	Felis catus	Viet Nam	2018	A
257	MT857284	F2	Felis catus	Viet Nam	2018	A
258	MT857285	F3	Felis catus	Viet Nam	2018	A

259	MT857286	F4	Felis catus	Viet Nam	2018	A
260	MT857268	F5	Felis catus	Viet Nam	2018	A
261	MT857269	F6	Felis catus	Viet Nam	2018	A
262	MT857270	F7	Felis catus	Viet Nam	2018	A
263	MT857271	F8	Felis catus	Viet Nam	2018	A
264	MT857272	F9	Felis catus	Viet Nam	2018	A
265	MK266783	Haerbin-01	Felis catus	China/Heilongjiang	2018	A
266	MK357741	HN10	Canis lupus familiaris	Viet Nam	2018	A
267	OQ868533	HN1801	Felis catus	China/Henan	2018	A
268	OQ868534	HN1802	Felis catus	China/Henan	2018	S
269	OQ868535	HN1803	Felis catus	China/Henan	2018	S
270	OQ868536	HN1804	Felis catus	China/Henan	2018	S
271	OQ868537	HN1805	Felis catus	China/Henan	2018	S
272	OQ868538	HN1806	Felis catus	China/Henan	2018	A
273	MK357740	HN40AA	Canis lupus familiaris	Viet Nam	2018	A
274	MK357742	HN41AA	Canis lupus familiaris	Viet Nam	2018	A

275	MK357743	HN7	Canis lupus familiaris	Viet Nam	2018	A	
276	MK266799	Jilin52	Felis catus	China/Jilin	2018	A	
277	OR260993	PSY01-2	Felis catus	China/Beijing	2018	A	
278	MZ442308	SMU-D14	Felis catus	China/Sichuan	2018	A	
279	MZ442310	SMU-D18	Felis catus	China/Sichuan	2018	A	
280	MZ442311	SMU-D28	Felis catus	China/Sichuan	2018	A	
281	MZ442305	SMU-D53	Felis catus	China/Sichuan	2018	A	
282	MK266793	Tanjin-01	Felis catus	China/Tianjin	2018	A	
283	MK266794	Tianjin-02	Felis catus	China/Tianjin	2018	A	
284	MN270937	VT2020	Canine	Thailand	2018	A	
285	MZ508522	18DIAPD52063/2	Felis catus	Italy	2018	S	
286	MZ508525	18DIAPD932/2	Felis catus	Italy	2018	S	
287	OQ815872	FPV-15	Felis catus	China/Jiangsu	2018	S	
288	MT274378	245-1478	Badger	Italy	2019	A	
289	OQ868553	AH1904	Felis catus	China/Anhui	2019	S	55/123 (45%)
290	MT078767	BCC1	Felis catus	India	2019	A	

291	MT270584	BJ016	Felis catus	China/Beijing	2019	A	
292	MT270581	BJ050	Felis catus	China/Beijing	2019	S	
293	MT270580	BJ051	Felis catus	China/Beijing	2019	S	
294	MT270579	BJ058	Felis catus	China/Beijing	2019	S	
295	MT270578	BJ061	Felis catus	China/Beijing	2019	A	
296	MT270576	BJ090	Felis catus	China/Beijing	2019	S	
297	MT270574	BJ128	Felis catus	China/Beijing	2019	S	
298	MT270571	BJ240	Felis catus	China/Beijing	2019	S	
299	MT270567	BJ318	Felis catus	China/Beijing	2019	A	
300	MT270566	BJ319	Felis catus	China/Beijing	2019	A	
301	MT270564	BJ372	Felis catus	China/Beijing	2019	A	
302	MT270563	BJ379	Felis catus	China/Beijing	2019	A	
303	MT270561	BJ405	Felis catus	China/Beijing	2019	S	
304	MT270557	BJ461	Felis catus	China/Beijing	2019	S	
305	MT270556	BJ481	Felis catus	China/Beijing	2019	S	
306	MT270554	BJ502	Felis catus	China/Beijing	2019	S	

307	MT270553	BJ540	Felis catus	China/Beijing	2019	S		
308	MT270550	BJ557	Felis catus	China/Beijing	2019	S		
309	MT270548	BJ565	Felis catus	China/Beijing	2019	S		
310	MT270541	BJ619	Felis catus	China/Beijing	2019	S		
311	MT270540	BJ624	Felis catus	China/Beijing	2019	S		
312	MT270531	BJ728	Felis catus	China/Beijing	2019	S		
313	ON646208	DL08	Felis catus	China/Liaoning	2019	S		
314	ON646209	DL09	Felis catus	China/Liaoning	2019	S		
315	ON646210	DL12	Felis catus	China/Liaoning	2019	A		
316	ON646211	DL13	Felis catus	China/Liaoning	2019	A		
317	ON646212	DL14	Felis catus	China/Liaoning	2019	A		
318	ON646213	DL15	Felis catus	China/Liaoning	2019	A		
319	ON646214	DL16	Felis catus	China/Liaoning	2019	A		
320	ON646215	DL17	Felis catus	China/Liaoning	2019	A		
321	ON646216	DL18	Felis catus	China/Liaoning	2019	A		
322	ON646217	DL19	Felis catus	China/Liaoning	2019	A		

323	ON646218	DL20	Felis catus	China/Liaoning	2019	A	
324	MN418997	DLC01	Felis catus	China/Liaoning	2019	A	
325	MN418998	DLC02	Felis catus	China/Liaoning	2019	A	
326	MN418999	DLC03	Felis catus	China/Liaoning	2019	S	
327	MN419000	DLC04	Felis catus	China/Liaoning	2019	A	
328	MN419001	DLC05	Felis catus	China/Liaoning	2019	A	
329	MN419002	DLC06	Felis catus	China/Liaoning	2019	A	
330	MN419003	DLC07	Felis catus	China/Liaoning	2019	A	
331	MN419004	DLC08	Felis catus	China/Liaoning	2019	A	
332	OP471918	HBSJZ19-01	Felis catus	China/Hebei	2019	S	
333	OQ868539	HN1901	Felis catus	China/Henan	2019	S	
334	OQ868540	HN1902	Felis catus	China/Henan	2019	S	
335	OQ868541	HN1903	Felis catus	China/Henan	2019	S	
336	OQ868542	HN1904	Felis catus	China/Henan	2019	S	
337	OQ868543	HN1905	Felis catus	China/Henan	2019	S	
338	OQ868544	HN1906	Felis catus	China/Henan	2019	A	

339	OQ868545	HN1908	Felis catus	China/Henan	2019	A	
340	OQ868546	HN1909	Felis catus	China/Henan	2019	A	
341	OQ868547	HN1910	Felis catus	China/Henan	2019	S	
342	MT892650	SX	Felis catus	China/Shaanxi	2019	S	
343	OP153928	19D01	Felis catus	South Korea	2019	A	
344	OP153929	19D02	Felis catus	South Korea	2019	A	
345	OP153930	19D03	Felis catus	South Korea	2019	A	
346	OP153931	19D04	Felis catus	South Korea	2019	A	
347	OP153932	19D05	Felis catus	South Korea	2019	A	
348	OQ868550	AH1901	Felis catus	China/Anhui	2019	S	
349	OQ868551	AH1902	Felis catus	China/Anhui	2019	S	
350	OQ868552	AH1903	Felis catus	China/Anhui	2019	S	
351	MT078768	BCC13	Felis catus	India	2019	A	
352	MT270585	BJ006	Felis catus	China/Beijing	2019	A	
353	MT270583	BJ020	Felis catus	China/Beijing	2019	A	
354	MT270582	BJ025	Felis catus	China/Beijing	2019	A	

355	MT270577	BJ078	Felis catus	China/Beijing	2019	A	
356	MT270575	BJ094	Felis catus	China/Beijing	2019	S	
357	MT270573	BJ133	Felis catus	China/Beijing	2019	S	
358	MT270572	BJ235	Felis catus	China/Beijing	2019	S	
359	MT270570	BJ277	Felis catus	China/Beijing	2019	A	
360	MT270569	BJ308	Felis catus	China/Beijing	2019	S	
361	MT270568	BJ309	Felis catus	China/Beijing	2019	S	
362	MT270565	BJ338	Felis catus	China/Beijing	2019	S	
363	MT270562	BJ396	Felis catus	China/Beijing	2019	S	
364	MT270560	BJ416	Felis catus	China/Beijing	2019	S	
365	MT270559	BJ435	Felis catus	China/Beijing	2019	S	
366	MT270558	BJ440	Felis catus	China/Beijing	2019	S	
367	MT270555	BJ501	Felis catus	China/Beijing	2019	S	
368	MT270552	BJ552	Felis catus	China/Beijing	2019	S	
369	MT270551	BJ554	Felis catus	China/Beijing	2019	S	
370	MT270549	BJ562	Felis catus	China/Beijing	2019	A	

371	MT270547	BJ572	Felis catus	China/Beijing	2019	S	
372	MT270546	BJ574	Felis catus	China/Beijing	2019	A	
373	MT270545	BJ577	Felis catus	China/Beijing	2019	A	
374	MT270544	BJ582	Felis catus	China/Beijing	2019	S	
375	MT270543	BJ588	Felis catus	China/Beijing	2019	A	
376	MT270542	BJ594	Felis catus	China/Beijing	2019	S	
377	MT270539	BJ625	Felis catus	China/Beijing	2019	S	
378	MT270538	BJ629	Felis catus	China/Beijing	2019	S	
379	MT270537	BJ638	Felis catus	China/Beijing	2019	S	
380	MT270536	BJ644	Felis catus	China/Beijing	2019	S	
381	OP471917	CC19-02	Felis catus	China/Jilin	2019	S	
382	MT078769	CPF6	Felis catus	India	2019	A	
383	MT078770	CV3	Felis catus	India	2019	A	
384	MN419007	DLC10	Felis catus	China/Liaoning	2019	A	
385	MN419008	DLC11	Felis catus	China/Liaoning	2019	A	
386	MN419009	DLC12	Felis catus	China/Liaoning	2019	A	

387	MN419010	DLC13	Felis catus	China/Liaoning	2019	A	
388	MN419011	DLC14	Felis catus	China/Liaoning	2019	A	
389	MN419012	DLC15	Felis catus	China/Liaoning	2019	S	
390	MN419013	DLC16	Felis catus	China/Liaoning	2019	A	
391	MN419005	DLC91	Felis catus	China/Liaoning	2019	A	
392	MN419006	DLC92	Felis catus	China/Liaoning	2019	A	
393	OM937916	EGY39-566	Felis catus	Egypt	2019	A	
394	MT857273	F10	Felis catus	Viet Nam	2019	A	
395	MT857274	F11	Felis catus	Viet Nam	2019	A	
396	MT857275	F12	Felis catus	Viet Nam	2019	A	
397	MT857276	F13	Felis catus	Viet Nam	2019	A	
398	MT857277	F14	Felis catus	Viet Nam	2019	A	
399	MT857278	F15	Felis catus	Viet Nam	2019	A	
400	MT857279	F16	Felis catus	Viet Nam	2019	A	
401	MT857280	F17	Felis catus	Viet Nam	2019	A	
402	MT857281	F18	Felis catus	Viet Nam	2019	A	

403	MT857282	F19	Felis catus	Viet Nam	2019	A	
404	OP471919	HBSJZ19-02	Felis catus	China/Hebei	2019	A	
405	MZ836347	JN-11	Felis catus	China/Shandong	2019	S	
406	MZ836378	JN-12	Felis catus	China/Shandong	2019	A	
407	MW017596	JSYZ-85	Canine	China/Jiangsu	2019	S	
408	MT078771	PDC3	Felis catus	India	2019	A	
409	MZ442304	SMU-D74	Felis catus	China/Sichuan	2019	A	
410	MZ442303	SMU-D87	Felis catus	China/Sichuan	2019	A	
411	OQ815870	FPV-6	Felis catus	China/Hubei	2020	S	
412	OQ868554	AH2001	Felis catus	China/Anhui	2020	S	
413	OQ868557	AH2004	Felis catus	China/Anhui	2020	S	
414	OQ868559	AH2007	Felis catus	China/Anhui	2020	S	
415	MT270532	BJ700	Felis catus	China/Beijing	2020	S	
416	MZ836377	JN-87	Felis catus	China/Shandong	2020	A	
417	MZ836376	JN-90	Felis catus	China/Shandong	2020	A	59/86 (67%)
418	MZ836375	JN-91	Felis catus	China/Shandong	2020	A	

419	MZ836374	JN-92	Felis catus	China/Shandong	2020	A
420	OQ868562	JS2001	Felis catus	China/Jiangsu	2020	S
421	MW017628	JSYZ-122	Felis catus	China/Jiangsu	2020	A
422	MW017629	JSYZ-123	Felis catus	China/Jiangsu	2020	S
423	MW017630	JSYZ-124	Felis catus	China/Jiangsu	2020	S
424	MZ391097	kucuk	Felis catus	Turkey	2020	A
425	OR551217	Luoyang-01	Felis catus	China/Henan	2020	S
426	OR551218	Luoyang-03	Felis catus	China/Henan	2020	S
427	MW017625	SH-118	Felis catus	China/Shanghai	2020	A
428	MW017626	SH-120	Felis catus	China/Shanghai	2020	S
429	MZ836370	TZ-104	Felis catus	China/Beijing	2020	A
430	MZ836369	TZ-108	Felis catus	China/Beijing	2020	A
431	MZ836368	TZ-112	Felis catus	China/Beijing	2020	S
432	MZ836366	TZ-122	Felis catus	China/Beijing	2020	A
433	MZ836365	TZ-124	Felis catus	China/Beijing	2020	A
434	MZ836364	TZ-133	Felis catus	China/Beijing	2020	S

435	MZ836363	TZ-135	Felis catus	China/Beijing	2020	S
436	MZ836362	TZ-148	Felis catus	China/Beijing	2020	A
437	MZ836361	TZ-185	Felis catus	China/Beijing	2020	S
438	MZ836360	TZ-193	Felis catus	China/Beijing	2020	A
439	MZ836358	TZ-235	Felis catus	China/Beijing	2020	S
440	MZ836357	TZ-236	Felis catus	China/Beijing	2020	A
441	MZ836356	TZ-237	Felis catus	China/Beijing	2020	S
442	MZ836355	TZ-238	Felis catus	China/Beijing	2020	S
443	MZ836354	TZ-239	Felis catus	China/Beijing	2020	S
444	MZ836353	TZ-240	Felis catus	China/Beijing	2020	S
445	MZ836352	TZ-241	Felis catus	China/Beijing	2020	S
446	MZ836351	TZ-242	Felis catus	China/Beijing	2020	A
447	MW495835	ZJFPV10	Felis catus	China/Zhejiang	2020	A
448	MW495836	ZJFPV11	Felis catus	China/Zhejiang	2020	S
449	MW495837	ZJFPV12	Felis catus	China/Zhejiang	2020	S
450	MW495838	ZJFPV13	Felis catus	China/Zhejiang	2020	A

45<br>1	MW49583<br>9	ZJFPV14	Felis catus	China/Zhejiang	2020	S
45<br>2	MW49584<br>0	ZJFPV15	Felis catus	China/Zhejiang	2020	S
45<br>3	MW49584<br>1	ZJFPV16	Felis catus	China/Zhejiang	2020	S
45<br>4	MW49584<br>2	ZJFPV17	Felis catus	China/Zhejiang	2020	S
45<br>5	MW49584<br>3	ZJFPV18	Felis catus	China/Zhejiang	2020	S
45<br>6	MW49584<br>4	ZJFPV19	Felis catus	China/Zhejiang	2020	S
45<br>7	MW49582<br>9	ZJFPV2	Felis catus	China/Zhejiang	2020	S
45<br>8	MW49584<br>5	ZJFPV20	Felis catus	China/Zhejiang	2020	S
45<br>9	MW49584<br>6	ZJFPV21	Felis catus	China/Zhejiang	2020	S
46<br>0	MW49584<br>7	ZJFPV22	Felis catus	China/Zhejiang	2020	S
46<br>1	MW49584<br>8	ZJFPV23	Felis catus	China/Zhejiang	2020	A
46<br>2	MW49583<br>0	ZJFPV4	Felis catus	China/Zhejiang	2020	A
46<br>3	MW49583<br>1	ZJFPV5	Felis catus	China/Zhejiang	2020	A
46<br>4	MW49583<br>2	ZJFPV6	Felis catus	China/Zhejiang	2020	S
46<br>5	MW49583<br>3	ZJFPV7	Felis catus	China/Zhejiang	2020	A
46<br>6	MW49583<br>4	ZJFPV8	Felis catus	China/Zhejiang	2020	A

467	MW017616	ZJHN-135	Canine	China/Zhejiang	2020	S
468	MW017618	ZJHN-138	Canine	China/Zhejiang	2020	S
469	OQ868555	AH2002	Felis catus	China/Anhui	2020	S
470	OQ868556	AH2003	Felis catus	China/Anhui	2020	S
471	OQ868558	AH2006	Felis catus	China/Anhui	2020	A
472	OQ868560	AH2009	Felis catus	China/Anhui	2020	S
473	OQ868563	AH2010	Felis catus	China/Anhui	2020	S
474	OQ868561	AH2011	Felis catus	China/Anhui	2020	S
475	OQ868564	AH2012	Felis catus	China/Anhui	2020	S
476	MZ391096	Barut	Felis catus	Turkey	2020	A
477	OQ868548	HB2001	Felis catus	China/Hubei	2020	S
478	OQ868549	HB2002	Felis catus	China/Hubei	2020	S
479	OQ868569	HB2003	Felis catus	China/Hubei	2020	S
480	MZ836373	JN-96	Felis catus	China/Shandong	2020	S
481	OQ868565	JS2002	Felis catus	China/Jiangsu	2020	S
482	MW791426	JSYZ-168	Felis catus	China/Jiangsu	2020	S

483	MW791427	JSYZ-169	Felis catus	China/Jiangsu	2020	S	
484	OR551219	Luoyang-08	Felis catus	China/Henan	2020	S	
485	OR551220	Luoyang-13	Felis catus	China/Henan	2020	A	
486	OR551221	Luoyang-19	Felis catus	China/Henan	2020	S	
487	MW017627	SH-121	Felis catus	China/Shanghai	2020	A	
488	MZ442302	SMU-F33	Felis catus	China/Sichuan	2020	S	
489	MZ442313	SMU-SC20-2	Felis catus	China/Sichuan	2020	S	
490	MZ442314	SMU-SC20-6	Felis catus	China/Sichuan	2020	S	
491	MZ836359	TZ-195	Felis catus	China/Beijing	2020	A	
492	MZ836350	TZ-243	Felis catus	China/Beijing	2020	S	
493	MZ836371	TZ-99	Felis catus	China/Beijing	2020	A	
494	OR551222	Zhengzhou-01	Felis catus	China/Henan	2020	S	
495	OR551224	Zhengzhou-11	Felis catus	China/Henan	2020	S	
496	MW017631	ZJHN-126	Felis catus	China/Zhejiang	2020	S	
497	PP738170	Cat-1	Felis catus	China/Henan	2021	A	60/89 (67%)
498	PP738171	Cat-2	Felis catus	China/Henan	2021	S	

499	PP738172	Cat-3	Felis catus	China/Henan	2021	S
500	PP738173	Cat-4	Felis catus	China/Henan	2021	A
501	PP738174	Cat-5	Felis catus	China/Henan	2021	A
502	ON185552	1724-HU	Felis catus	Hungary	2021	A
503	OQ718429	IT	Felis catus	Italy	2021	A
504	OR227624	CH/YCYH/2021	Felis catus	China/Jiangxi	2021	S
505	OR551227	Anyang-02	Felis catus	China/Henan	2021	S
506	MT270533	BJ698	Felis catus	China/Beijing	2021	S
507	OQ398386	FPV003	Felis catus	China/Shandong	2021	A
508	OQ398387	FPV008	Felis catus	China/Shandong	2021	A
509	OQ398388	FPV013	Felis catus	China/Shandong	2021	A
510	OQ398389	FPV014	Felis catus	China/Shandong	2021	A
511	OQ398390	FPV021	Felis catus	China/Shandong	2021	A
512	OQ398391	FPV026	Felis catus	China/Shandong	2021	A
513	OQ398392	FPV027	Felis catus	China/Shandong	2021	A
514	OQ398394	FPV029	Felis catus	China/Shandong	2021	S

515	OQ398395	FPV030	Felis catus	China/Shandong	2021	S
516	OQ398396	FPV036	Felis catus	China/Shandong	2021	S
517	OQ398398	FPV038	Felis catus	China/Shandong	2021	S
518	OQ398399	FPV039	Felis catus	China/Shandong	2021	S
519	OQ398401	FPV041	Felis catus	China/Shandong	2021	S
520	OQ398402	FPV042	Felis catus	China/Shandong	2021	S
521	OQ398403	FPV043	Felis catus	China/Shandong	2021	S
522	OQ398418	FPV046	Felis catus	China/Shandong	2021	A
523	OQ398419	FPV048	Felis catus	China/Shandong	2021	A
524	OQ398406	FPV072	Felis catus	China/Shandong	2021	S
525	OQ398408	FPV077	Felis catus	China/Shandong	2021	S
526	OQ398409	FPV080	Felis catus	China/Shandong	2021	S
527	OQ398413	FPV086	Felis catus	China/Shandong	2021	S
528	OP796708	JSNJ-21G4	Felis catus	China/Jiangsu	2021	S
529	OP796709	JSNJ-21G5	Felis catus	China/Jiangsu	2021	S
530	OP796706	SH-21D2	Felis catus	China/Shanghai	2021	S

531	OQ615261	UFUUSP15	Felis catus	Brazil	2021	A
532	OQ615262	UFUUSP16	Felis catus	Brazil	2021	A
533	OQ615263	UFUUSP17	Felis catus	Brazil	2021	A
534	OM885379	Yanji10	Felis catus	China/Jilin	2021	S
535	OM885380	Yanji11	Felis catus	China/Jilin	2021	S
536	OM885381	Yanji12	Felis catus	China/Jilin	2021	A
537	OM885382	Yanji13	Felis catus	China/Jilin	2021	S
538	OM885383	Yanji15	Felis catus	China/Jilin	2021	S
539	OM885384	Yanji18	Felis catus	China/Jilin	2021	S
540	OM918770	Yanji23	Felis catus	China/Jilin	2021	A
541	OM918780	Yanji35	Felis catus	China/Jilin	2021	S
542	OM885377	Yanji8	Felis catus	China/Jilin	2021	L
543	OM885378	Yanji9	Felis catus	China/Jilin	2021	S
544	OQ398393	FPV028	Felis catus	China/Shandong	2021	A
545	OQ398397	FPV037	Felis catus	China/Shandong	2021	S
546	OQ398400	FPV040	Felis catus	China/Shandong	2021	S

547	OQ398404	FPV044	Felis catus	China/Shandong	2021	S
548	OQ398405	FPV045	Felis catus	China/Shandong	2021	S
549	OQ398420	FPV049	Felis catus	China/Shandong	2021	A
550	OQ398421	FPV050	Felis catus	China/Shandong	2021	A
551	OQ398422	FPV055	Felis catus	China/Shandong	2021	S
552	OQ398407	FPV076	Felis catus	China/Shandong	2021	S
553	OQ398410	FPV081	Felis catus	China/Shandong	2021	S
554	OQ398411	FPV082	Felis catus	China/Shandong	2021	S
555	OQ398412	FPV085	Felis catus	China/Shandong	2021	S
556	OQ398414	FPV087	Felis catus	China/Shandong	2021	S
557	OQ398415	FPV088	Felis catus	China/Shandong	2021	S
558	OQ398416	FPV089	Felis catus	China/Shandong	2021	S
559	OQ398417	FPV090	Felis catus	China/Shandong	2021	S
560	OQ868566	HN2101	Felis catus	China/Henan	2021	S
561	OQ868567	HN2105	Felis catus	China/Henan	2021	S
562	OR194141	SDYT2	Felis catus	China/Shandong	2021	S

563	OP796707	SH-21D4	Felis catus	China/Shanghai	2021	S
564	OM212011	Yanji	Felis catus	China/Jilin	2021	S
565	OM918783	Yanji17	Felis catus	China/Jilin	2021	S
566	OM322821	Yanji2	Felis catus	China/Jilin	2021	A
567	OM918771	Yanji24	Felis catus	China/Jilin	2021	S
568	OM918772	Yanji25	Felis catus	China/Jilin	2021	S
569	OM918784	Yanji26	Felis catus	China/Jilin	2021	S
570	OM918773	Yanji27	Felis catus	China/Jilin	2021	A
571	OM918774	Yanji28	Felis catus	China/Jilin	2021	S
572	OM918775	Yanji29	Felis catus	China/Jilin	2021	S
573	OM885373	Yanji3	Felis catus	China/Jilin	2021	S
574	OM918776	Yanji30	Felis catus	China/Jilin	2021	L
575	OM918777	Yanji31	Felis catus	China/Jilin	2021	S
576	OM918778	Yanji32	Felis catus	China/Jilin	2021	S
577	OM918779	Yanji33	Felis catus	China/Jilin	2021	S
578	OM918781	Yanji36	Felis catus	China/Jilin	2021	A

579	OM918785	Yanji37	Felis catus	China/Jilin	2021	S		
580	OM918782	Yanji38	Felis catus	China/Jilin	2021	S		
581	OM885374	Yanji4	Felis catus	China/Jilin	2021	S		
582	OM885375	Yanji5	Felis catus	China/Jilin	2021	A		
583	OM885376	Yanji6	Felis catus	China/Jilin	2021	S		
584	OR551223	Zhengzhou-07	Felis catus	China/Henan	2021	S		
585	OR551225	Zhengzhou-26	Felis catus	China/Henan	2021	A		
586	OR211672	AHWH55	Felis catus	China/Anhui	2022	A		
587	MT270534	BJ663	Felis catus	China/Beijing	2022	A		
588	OR399569	FPV109	Felis catus	China/Jiangsu	2022	S		
589	OR399570	FPV111	Felis catus	China/Jiangsu	2022	S		
590	OR399559	FPV13	Felis catus	China/Jiangsu	2022	S		75/87 (86%)
591	OR399571	FPV132	Felis catus	China/Jiangsu	2022	S		
592	OR399572	FPV133	Felis catus	China/Jiangsu	2022	S		
593	OR399573	FPV141	Felis catus	China/Jiangsu	2022	S		
594	OR399574	FPV143	Felis catus	China/Jiangsu	2022	S		

595	OR399575	FPV149	Felis catus	China/Jiangsu	2022	S
596	OR399560	FPV19	Felis catus	China/Jiangsu	2022	S
597	OR399565	FPV19	Felis catus	China/Jiangsu	2022	S
598	OR399561	FPV34	Felis catus	China/Jiangsu	2022	S
599	OR399562	FPV41	Felis catus	China/Jiangsu	2022	S
600	OR399563	FPV42	Felis catus	China/Jiangsu	2022	S
601	OR399564	FPV56	Felis catus	China/Jiangsu	2022	S
602	OR399566	FPV73	Felis catus	China/Jiangsu	2022	S
603	OR399567	FPV77	Felis catus	China/Jiangsu	2022	S
604	OR399568	FPV89	Felis catus	China/Jiangsu	2022	S
605	OQ868568	JS2201	Felis catus	China/Jiangsu	2022	S
606	OR194110	JSZJ1	Felis catus	China/Jiangsu	2022	S
607	OQ570642	JZ2022	Felis catus	China/Liaoning	2022	S
608	OQ535501	SDQD14	Felis catus	China/Shandong	2022	S
609	OR194121	SH1	Felis catus	China/Shanghai	2022	S
610	OR194124	SH4	Felis catus	China/Shanghai	2022	S

611	OR194125	SH5	Felis catus	China/Shanghai	2022	S
612	OP796714	ZJHN-2206	Felis catus	China/Zhejiang	2022	A
613	OP796716	ZJHN-2208	Felis catus	China/Zhejiang	2022	A
614	OR211675	AHWH52	Felis catus	China/Anhui	2022	S
615	OR211674	AHWH53	Felis catus	China/Anhui	2022	S
616	OR211673	AHWH54	Felis catus	China/Anhui	2022	S
617	OR194142	AHWH56	Felis catus	China/Anhui	2022	S
618	OR194133	FJFZ1	Felis catus	China/Fujian	2022	S
619	OR194134	FJFZ2	Felis catus	China/Fujian	2022	S
620	OR194135	FJFZ3	Felis catus	China/Fujian	2022	S
621	OR211676	FJFZ4	Felis catus	China/Fujian	2022	S
622	OR194129	GDGZ1	Felis catus	China/Guangdong	2022	S
623	OR194130	GDGZ2	Felis catus	China/Guangdong	2022	S
624	OR194131	GDGZ3	Felis catus	China/Guangdong	2022	S
625	OR194132	GDGZ4	Felis catus	China/Guangdong	2022	S
626	OR194126	HNZZ1	Felis catus	China/Henan	2022	S

627	OR194127	HNZZ2	Felis catus	China/Henan	2022	S
628	OR194128	HNZZ3	Felis catus	China/Henan	2022	S
629	OR194136	JSWX1	Felis catus	China/Jiangsu	2022	S
630	OR194137	JSWX2	Felis catus	China/Jiangsu	2022	S
631	OR194138	JSWX3	Felis catus	China/Jiangsu	2022	S
632	OR194139	JSWX4	Felis catus	China/Jiangsu	2022	S
633	OR194140	JSWX5	Felis catus	China/Jiangsu	2022	S
634	OR194111	JSZJ2	Felis catus	China/Jiangsu	2022	S
635	OQ869254	LZ05	Canine	China/Gansu	2022	S
636	OR783313	LZ092022	Felis catus	China/Gansu	2022	S
637	ON605652	RCP vaccine	Felis catus	Australia	2022	A
638	OQ535504	SDQD21	Felis catus	China/Shandong	2022	S
639	OQ535505	SDQD23	Felis catus	China/Shandong	2022	S
640	OQ535496	SDQD6	Felis catus	China/Shandong	2022	S
641	OQ535507	SDYT22	Felis catus	China/Shandong	2022	A
642	OR211671	SDYT3	Felis catus	China/Shandong	2022	A

643	OR194122	SH2	Felis catus	China/Shanghai	2022	S
644	OR194123	SH3	Felis catus	China/Shanghai	2022	S
645	OR551226	Xinxiang-05	Felis catus	China/Henan	2022	S
646	OP796713	ZJHN-2205	Felis catus	China/Zhejiang	2022	A
647	OP796715	ZJHN-2207	Felis catus	China/Zhejiang	2022	S
648	OP796710	ZJHZ-2202	Felis catus	China/Zhejiang	2022	S
649	OP796711	ZJHZ-2203	Felis catus	China/Zhejiang	2022	S
650	OP796712	ZJHZ-2204	Felis catus	China/Zhejiang	2022	S
651	OR399559	13	Felis catus	China/Jiangsu	2022	S
652	OR399560	19	Felis catus	China/Jiangsu	2022	S
653	OR399561	34	Felis catus	China/Jiangsu	2022	S
654	OR399562	41	Felis catus	China/Jiangsu	2022	S
655	OR399563	42	Felis catus	China/Jiangsu	2022	S
656	OR399564	56	Felis catus	China/Jiangsu	2022	S
657	OR399565	59	Felis catus	China/Jiangsu	2022	S
658	OR399566	73	Felis catus	China/Jiangsu	2022	S

659	OR399567	77	Felis catus	China/Jiangsu	2022	S	
660	OR399568	89	Felis catus	China/Jiangsu	2022	S	
661	OR709671	QD22-1	Felis catus	China/Shandong	2022	S	
662	OR727315	QD22-5	Felis catus	China/Shandong	2022	S	
663	OR727316	QD22-4	Felis catus	China/Shandong	2022	S	
664	OR727317	QD22-8	Felis catus	China/Shandong	2022	A	
665	OR727318	QD22-3	Felis catus	China/Shandong	2022	S	
666	OR727319	QD22-13	Felis catus	China/Shandong	2022	S	
667	PP035815	MZ26	Felis catus	India	2022	S	
668	PP035816	MZ33	Felis catus	India	2022	S	
669	PP035817	MZ35	Felis catus	India	2022	S	
670	PP419033	MZ27	Felis catus	India	2022	A	
671	PP419034	MZ29	Felis catus	India	2022	A	
672	PP419035	MZ30	Felis catus	India	2022	A	
673	OR365078	KTPV-2305	Siberian tiger	South Korea	2023	A	45/101<br>21/25, (84%), China<br>24/37, (65%), Viet Nam
674	MT270535	BJ662	Felis catus	China/Beijing	2023	S	

675	OQ615264	Nobivac vaccine strain	Felis catus	Brazil	2023	A
676	PP781541	CTU-AG1	Felis catus	Viet Nam	2023	S
677	PP781542	CTU-AG2	Felis catus	Viet Nam	2023	S
678	PP781543	CTU-AG3	Felis catus	Viet Nam	2023	S
679	PP781544	CTU-AG4	Felis catus	Viet Nam	2023	S
680	PP781545	CTU-AG5	Felis catus	Viet Nam	2023	S
681	PP781546	CTU-AG6	Felis catus	Viet Nam	2023	S
682	PP781547	CTU-AG7	Felis catus	Viet Nam	2023	S
683	PP781548	CTU-AG8	Felis catus	Viet Nam	2023	S
684	PP781533	CTU-CM1	Felis catus	Viet Nam	2023	A
685	PP781534	CTU-CM2	Felis catus	Viet Nam	2023	S
686	PP781535	CTU-CM3	Felis catus	Viet Nam	2023	A
687	PP781536	CTU-CM4	Felis catus	Viet Nam	2023	A
688	PP781537	CTU-CM5	Felis catus	Viet Nam	2023	A
689	PP781538	CTU-CM6	Felis catus	Viet Nam	2023	A
690	PP781539	CTU-CM7	Felis catus	Viet Nam	2023	S

691	PP781540	CTU-CM8	Felis catus	Viet Nam	2023	S
692	PP663044	FVMCU 8	Felis catus	Egypt	2023	A
693	PP663045	FVMCU (10)	Felis catus	Egypt	2023	A
694	PP663046	FVMCU (12)	Felis catus	Egypt	2023	A
695	PP663047	FVMCU (16)	Felis catus	Egypt	2023	A
696	PP663048	FVMCU (17)	Felis catus	Egypt	2023	A
697	PP663049	FVMCU (18)	Felis catus	Egypt	2023	A
698	PP663050	FVMCU (20)	Felis catus	Egypt	2023	A
699	PP663051	FVMCU (21)	Felis catus	Egypt	2023	A
700	PP663052	FVMCU (22)	Felis catus	Egypt	2023	A
701	PP663053	FVMCU (25)	Felis catus	Egypt	2023	A
702	PP663054	FVMCU (26)	Felis catus	Egypt	2023	A
703	PP663055	FVMCU (27)	Felis catus	Egypt	2023	A
704	PP663056	FVMCU (28)	Felis catus	Egypt	2023	A
705	PP663057	FVMCU (29)	Felis catus	Egypt	2023	A
706	PP663058	FVMCU (30)	Felis catus	Egypt	2023	A

70 7	PP663059	FVMCU (31)	Felis catus	Egypt	2023	A
70 8	PP663060	FVMCU (32)	Felis catus	Egypt	2023	A
70 9	PP663061	FVMCU (44)	Felis catus	Egypt	2023	A
71 0	PP663062	FVMCU (51)	Felis catus	Egypt	2023	A
711	PP663063	FVMCU (53)	Felis catus	Egypt	2023	A
71 2	PP663064	FVMCU (59)	Felis catus	Egypt	2023	A
71 3	PP663065	FVMCU (70)	Felis catus	Egypt	2023	A
71 4	PP663066	FVMCU (71)	Felis catus	Egypt	2023	A
71 5	PP663067	FVMCU (81)	Felis catus	Egypt	2023	A
71 6	PP663068	FVMCU (82)	Felis catus	Egypt	2023	A
71 7	PP663069	FVMCU (90)	Felis catus	Egypt	2023	A
71 8	PP663070	FVMCU (100)	Felis catus	Egypt	2023	A
71 9	PP663071	FVMCU (102)	Felis catus	Egypt	2023	A
72 0	PP663072	FVMCU (103)	Felis catus	Egypt	2023	A
72 1	PP663073	FVMCU (104)	Felis catus	Egypt	2023	A
72 2	PP781517	CTU-CT01	Felis catus	Viet Nam	2023	S

723	PP781518	CTU-CT02	Felis catus	Viet Nam	2023	S
724	PP781519	CTU-CT03	Felis catus	Viet Nam	2023	S
725	PP781520	CTU-CT04	Felis catus	Viet Nam	2023	A
726	PP781521	CTU-CT05	Felis catus	Viet Nam	2023	S
727	PP781522	CTU-CT06	Felis catus	Viet Nam	2023	S
728	PP781523	CTU-CT07	Felis catus	Viet Nam	2023	S
729	PP781524	CTU-CT08	Felis catus	Viet Nam	2023	S
730	PP781525	CTU-TV01	Felis catus	Viet Nam	2023	S
731	PP781526	CTU-TV02	Felis catus	Viet Nam	2023	A
732	PP781527	CTU-TV03	Felis catus	Viet Nam	2023	S
733	PP781528	CTU-TV04	Felis catus	Viet Nam	2023	S
734	PP781529	CTU-TV05	Felis catus	Viet Nam	2023	S
735	PP781530	CTU-TV06	Felis catus	Viet Nam	2023	S
736	PP781531	CTU-TV07	Felis catus	Viet Nam	2023	S
737	PP781532	CTU-TV08	Felis catus	Viet Nam	2023	A
738	OR652079	CTU/FVM-TV005	Felis catus	Viet Nam	2023	A

739	OR652080	CTU/FVM-TV004	Felis catus	Viet Nam	2023	A
740	OR652081	CTU/FVM-TV003	Felis catus	Viet Nam	2023	A
741	OR652082	CTU/FVM-TV002	Felis catus	Viet Nam	2023	A
742	OR652083	CTU/FVM-TV001	Felis catus	Viet Nam	2023	A
743	OR399569	FPV-109	Felis catus	China/Jiangsu	2023	A
744	OR399570	FPV-111	Felis catus	China/Jiangsu	2023	A
745	OR399571	FPV-132	Felis catus	China/Jiangsu	2023	A
746	OR399572	FPV-133	Felis catus	China/Jiangsu	2023	S
747	OR399573	FPV-141	Felis catus	China/Jiangsu	2023	S
748	OR399574	FPV-143	Felis catus	China/Jiangsu	2023	S
749	OR399575	FPV-149	Felis catus	China/Jiangsu	2023	S
750	PP619442	F6-2/NJ2304	Felis catus	China/Jiangsu	2023	S
751	PP619441	F24-1/YZ2309	Felis catus	China/Jiangsu	2023	S
752	PP619440	F22-1/YZ2309	Felis catus	China/Jiangsu	2023	S
753	PP619439	F16-1/NJ2307	Felis catus	China/Jiangsu	2023	S
754	PP619437	F11-1/NJ2304	Felis catus	China/Jiangsu	2023	S

755	PP619436	F10-1/NJ2304	Felis catus	China/Jiangsu	2023	S	
756	PP619435	F2-1/NJ2304	Felis catus	China/Jiangsu	2023	S	
757	PP619438	F14-1/HZ2304	Felis catus	China/Zhejiang	2023	S	
758	PP336908	CVASU/BD/50	Felis catus	Bangladesh	2023	A	
759	PP738175	Cat-6	Felis catus	Chin/Xinjiang	2023	S	
760	PQ212863	XJ-SHZ-1	Felis catus	Chin/Xinjiang	2023	S	
761	PQ212864	XJ-SHZ-3	Felis catus	Chin/Xinjiang	2023	S	
762	PQ212865	XJ-SHZ-4	Felis catus	Chin/Xinjiang	2023	S	
763	PQ212866	XJ-URC-5	Felis catus	Chin/Xinjiang	2023	S	
764	PQ212867	XJ-URC-6	Felis catus	Chin/Xinjiang	2023	S	
765	PQ212868	XJ-URC-7	Felis catus	Chin/Xinjiang	2023	S	
766	PQ212869	XJ-URC-8	Felis catus	Chin/Xinjiang	2023	A	
767	PQ227071	XJ-SHZ-2	Felis catus	Chin/Xinjiang	2023	S	
768	OR602718	ITA/2023/hystrix/213	Hystrix cristata	Italy	2023	A	
769	OR602717	ITA/2023/bear/74	Ursus arctos marsicanus	Italy	2023	A	
770	PP663074	FVMCU (107)	Felis catus	Egypt	2024	A	

Figure S1Immunochromatographic strip detect of FPLV shedding in faeces

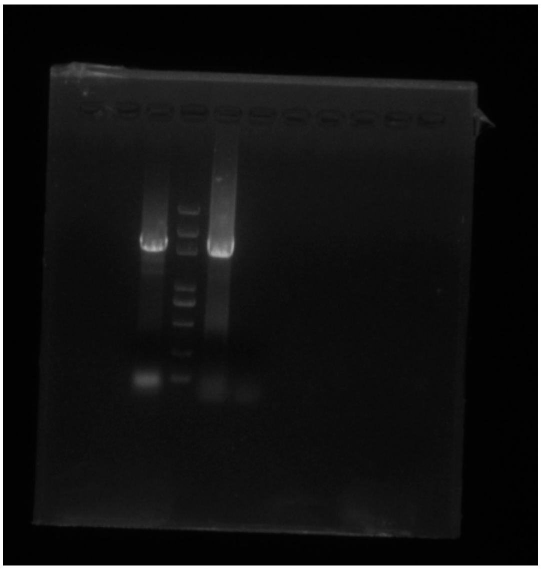

Original picture of figure 1C
